# Characterization of the genome of bald cypress

**DOI:** 10.1186/1471-2164-12-553

**Published:** 2011-11-11

**Authors:** Wenxuan Liu, Supaphan Thummasuwan, Sunish K Sehgal, Philippe Chouvarine, Daniel G Peterson

**Affiliations:** 1 Mississippi Genome Exploration Laboratory and Department of Plant & Soil Sciences, Mississippi State University, Mississippi State, MS 39762, USA; 2 Department of Plant Pathology, Kansas State University, Manhattan, KS 66506, USA; 3 Institute for Genomics, Biocomputing & Biotechnology, Mississippi State, MS 39762, USA

## Abstract

**Background:**

Bald cypress (*Taxodium distichum var. distichum*) is a coniferous tree of tremendous ecological and economic importance. It is a member of the family Cupressaceae which also includes cypresses, redwoods, sequoias, thujas, and junipers. While the bald cypress genome is more than three times the size of the human genome, its 1C DNA content is amongst the smallest of any conifer. To learn more about the genome of bald cypress and gain insight into the evolution of Cupressaceae genomes, we performed a Cot analysis and used Cot filtration to study *Taxodium *DNA. Additionally, we constructed a 6.7 genome-equivalent BAC library that we screened with known *Taxodium *genes and select repeats.

**Results:**

The bald cypress genome is composed of 90% repetitive DNA with most sequences being found in low to mid copy numbers. The most abundant repeats are found in fewer than 25,000 copies per genome. Approximately 7.4% of the genome is single/low-copy DNA (i.e., sequences found in 1 to 5 copies). Sequencing of highly repetitive Cot clones indicates that most *Taxodium *repeats are highly diverged from previously characterized plant repeat sequences. The bald cypress BAC library consists of 606,336 clones (average insert size of 113 kb) and collectively provides 6.7-fold genome equivalent coverage of the bald cypress genome. Macroarray screening with known genes produced, on average, about 1.5 positive clones per probe per genome-equivalent. Library screening with Cot-1 DNA revealed that approximately 83% of BAC clones contain repetitive sequences iterated 10^3 ^to 10^4 ^times per genome.

**Conclusions:**

The BAC library for bald cypress is the first to be generated for a conifer species outside of the family Pinaceae. The *Taxodium *BAC library was shown to be useful in gene isolation and genome characterization and should be an important tool in gymnosperm comparative genomics, physical mapping, genome sequencing, and gene/polymorphism discovery. The single/low-copy (SL) component of bald cypress is 4.6 times the size of the *Arabidopsis *genome. As suggested for other gymnosperms, the large amount of SL DNA in *Taxodium *is likely the result of divergence among ancient repeat copies and gene/pseudogene duplication.

## Background

The conifer family Cupressaceae contains many remarkable and important trees including junipers, redwoods, sequoias, cypresses, and thujas [1]. One Cupressaceae species that is of tremendous ecological importance to the southeastern U.S. is bald cypress, *Taxodium distichum *(L.) Rich var. *distichum *[2]. Bald cypress is the cornerstone species in the aptly named "cypress swamps" where it serves as a source of food and shelter for numerous and sundry organisms [3]. Though native to the U.S. South, bald cypress is a popular ornamental throughout much of the world; indeed, it has been cultivated in Europe since at least the mid 17^th ^Century [4]. Bald cypress wood is extremely resistant to wind, water, pathogens, and pests, something that perhaps is not surprising when one considers that individual trees may spend their entire life (sometimes > 1500 years - [5]) partially submerged in water. The highly durable wood of bald cypress is used in construction of boats, docks, bridges, and roofing shingles [6], although the tree's relatively slow growth-rate has limited its use as a wood crop. Unlike most conifers, bald cypress is deciduous with leaves that change from light green to brown in the fall. Its attractive appearance and hardiness have made it a popular ornamental throughout the eastern U.S. [2].

The genus *Taxodium *consists of one to three extant species, depending upon taxonomic preference. The most conservative treatment places all trees in a single species (*T. distichum*) with three varieties; specifically bald cypress (*T. distichum *var. *distichum*), pond cypress (*T. distichum *var. *imbricarium*), and Montezuma bald cypress (*T. distichum *var. *mexicanum*). While the single species treatment is phylogenetically warranted [7-9], sociological reasons have kept the multi-species nomenclature in place - e.g., Montezuma bald cypress is the national tree of Mexico [10].

The bald cypress 1C DNA content is 9731 Mb [11] which places it amongst the smallest of conifer genomes [12]. Bald cypress possesses 2*n *= 2*x *= 22 chromosomes [13] and is a diploid like most members of the Cupressaceae [12].

*Taxodium *has not been the subject of molecular mapping and/or EST sequencing. In the Cupressaceae, molecular research has largely focused on *Cryptomeria japonica*, and molecular maps based upon EST, RFLP, RAPD, and isozyme markers exist for this species [14-16]. However, these genetic maps cover only a small part of the entire *Cryptomeria *genome.

To advance understanding of *Taxodium *and the Cupressaceae in general, we utilized three experimental tools. Specifically:

(1) *Cot analysis *- The study of DNA reassociation kinetics in solution is known as Cot analysis [17]. It is one of the earliest means of studying genome structure predating cloning and DNA sequencing techniques by several years (see 18 for review). Cot analysis is based upon the observation that the product of DNA concentration (*C_0_*), reassociation time (*t*), and a "buffer factor" accounting for cation concentration (*δ*) has a predictable effect on the amount of reassociation occurring in a denatured DNA sample [18]. The major unknown factor influencing reassociation is the underlying sequence composition of the DNA. Consequently, one can indirectly study genome sequence composition by exploring how changes in *C_0_tδ *(known by the colloquialism "Cot") influence reassociation. Typically, a graph is created in which the fraction of reassociated DNA is plotted against the logarithm of Cot (from Cot ~ 0 to Cot values at which reassociation is essentially complete). The resulting scatter plot is analyzed using nonlinear regression analysis, and a least-squares curve is fit through the data. This graph, known as a Cot curve, provides a visual representation of the genome. Analysis of Cot data provides the number of kinetic components in a genome, the reassociation rate (*k*) of each component, the fraction of the genome found in each component, the kinetic complexity (i.e., estimated sequence complexity) of each component, and each component's average sequence iteration. Additionally, in some instances the genome size of an organism can also be estimated through comparison of the *k *value for the single/low copy component of the organism of interest with the *k *and genome size of *E. coli *[19].

(2) *Cot filtration *(CF) - CF represents a merger between Cot analysis and high-throughput DNA sequencing [19-21]. In short, the results of a Cot curve are used to guide fractionation of a genome into its kinetic components, and isolated components are sequenced in full or part. The value of CF and other reduced-representation sequencing techniques lies in their ability to enrich for subsets of genomic DNA of interest [19,22]. Since the majority of genes are single/low-copy in nature, CF has been used to enrich for gene space including the promoters and introns missed by cDNA approaches [20,23,24]. Alternatively, sequencing of a highly repetitive component represents a means of efficiently exploring the repetitive landscape of a genome [25].

(3) *BAC library analysis *- Bacterial artificial chromosomes (BACs) have been the most popular large-insert cloning vectors for nearly 20 years [26], and ordered BAC libraries (i.e., libraries in which clone is individually archived) remain highly useful tools in modern genomics research [27]. One can efficiently map molecular markers to corresponding BACs via multiplex macroarray hybridization techniques or by using multiplex PCR strategies [28-30]. By combining macroarray/PCR mapping data with data from BAC end sequencing, DNA fingerprinting, and/or sequencing of BAC pools, one can generate highly accurate physical (DNA sequence) maps and identify minimum BAC tiling paths representing whole or nearly whole chromosomes [27]. Though construction of BAC minimum tiling paths will likely become less important as DNA sequencing becomes cheaper and faster, BAC libraries will likely remain a key means of bridging gaps and resolving anomalies in shotgun sequence-based scaffolds [31].

The bald cypress BAC library generated in this study affords 6.7 genome equivalent coverage of the bald cypress genome. Though BAC libraries exist for several Pinaceae conifers [31-33], to our knowledge the bald cypress BAC library is the first constructed for a Cupressaceae species. The library was shown to be useful in gene isolation and genome characterization. The Cot and CF-based repeat analyses suggest that highly diverged, low-copy repeats account for much of bald cypress' genomic DNA.

## Results

### Cot analysis

**Figure 1:**
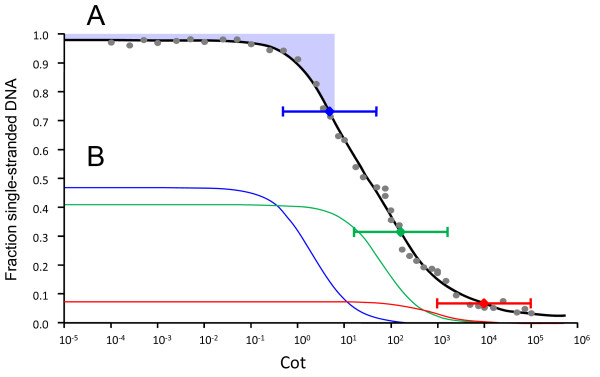
**Cot curve for bald cypress**. (A) A least-squares curve (black line) was fit through the data points (gray circles) using the CotQuest program of Bunge et al. [34]. The curve consists of highly repetitive (HR), moderately repetitive (MR) and single/low copy (SL) components, characterized by fast, intermediate, and slow reassociation, respectively. Blue, green, and red diamonds mark the Cot1/2 values of the HR, MR, and SL components. The brackets centered at a particular Cot1/2 marker show the "two Cot decade region" in which 80% of the sequences in that component will renature [63]. The blue shaded region at the top of the curve shows the double-stranded HR DNA (plus foldback sequences) isolated for sequencing. Biological data obtained from curve analysis is shown in Table 1. (B) The predicted individual renaturation profiles of the HR component, MR component, and SL component are shown.

The CotQuest [34] nonlinear regression model providing the best fit of the renaturation kinetics data was a three-component fit in which outliers had been removed (using CotQuest's built in outlier detection) and the reassociation rate (*k*) of the slowest reassociating component had been fixed based upon the genome size of bald cypress. This best fit Cot curve is shown in Figure 1 while the major biological characteristics obtained from curve analysis are shown in Table 1. Of note, the curve is composed of highly repetitive (HR), moderately repetitive (MR), and single/low-copy (SL) components accounting for 47.0, 41.1, and 7.4% of the genome, respectively. Because the best fit was obtained when the reassociation rate of the SL component was fixed based upon genome size, the curve cannot be used to produce an estimate of DNA content. Assuming that the SL component has a repetition frequency of 1, the average repetition frequency of the DNA in other components can be estimated by dividing their *k *values by the *k *value of the SL component [35]. The mean predicted repetition frequencies of sequences in the MR and HR components are 61 and 2054, respectively.

**Table 1:** Results of bald cypress Cot curve analysis

Component	Fraction of genome	KnCx^a ^(Mb)	*k *(M^-1^·s^-1^)	Cot1/2 (M·s)	MRF^b ^
HR	0.4702	0.2126	0.2157	4.64	2054
MR	0.4114	65.55	0.00637	56.99	61
SL	0.0744	719.2	0.000105	9523.81	1

**Other**					

Foldback	0.0174				
Unreassociated	0.0233				

^a^KnCx = kinetic complexity

^b^MRF = mean repetition frequency

For each component, 80% of the sequences in that component will reassociate in the "two Cot decade region" (TCDR) flanking the component's Cot1/2 value, i.e., if a component's Cot1/2 is *y*, then 80% of sequences can be found between 0.1*y *and 10*y*. Because *k *(and hence Cot1/2 which is the inverse of *k*) can be directly related to sequence copy number, one can use the TCDR to predict the range in sequence iteration for 80% of the sequences in a particular component. For example, the bald cypress MR component has a mean repeat frequency of 61 while 80% of the MR sequences are repeated from 6.1 to 610 times. Likewise, for the HR component which has a mean repetition frequency of 2054, 80% of elements in the component have iteration frequencies between 205.4 and 20,540.

### Cot filtration

**Figure 2:**
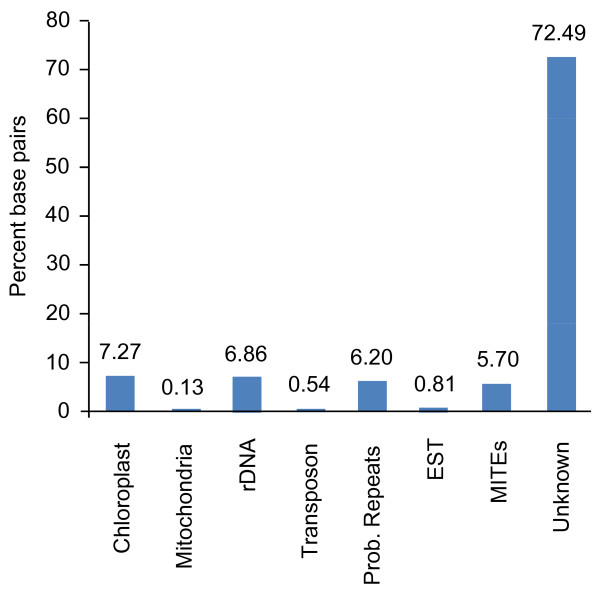
**Categorization of bald cypress HR sequences**. The HR sequence reads were classified using the Sequence Read Classification Pipeline of Chouvarine et al. [36] with minor modifications detailed in the Materials and Methods. Note that the majority of reads (72.49%) show no significant to homology to known gene or repeat sequences, nor are they categorized as "Probable Repeats" by the SRCP's *ab initio *repeat analysis functions.

743 high-quality HR capillary sequence reads were analyzed using the Sequence Read Classification Pipeline (SRCP) of Chouvarine et al. [36]. As shown in Figure 2, the vast majority of reads (72.49%) showed no obvious (S' ≥ 60) homology to previously characterized repeat sequences, gene sequences, and/or each other. Chloroplast DNA, rDNA, and "Probable Repeats" (i.e., sequences that appear to be repetitive based upon their relative frequency in the HR sequence set) each account for about 6-8% of the HR reads. Only 0.54% of sequenced HR sequences shared significant sequence identity with known mobile elements.

The program MUST [37] was used to identify potential miniature inverted-repeat transposable elements (MITEs) in the HR sequences. MITEs are non-autonomous DNA elements characterized by terminal-inverted repeats, target-site duplications (direct repeats), and an internal region with no coding sequence [38]. Those potential MITEs found in sequence reads classified as "Unknown" or "Probable Repeats" by the SRCP are provided in Additional file 1. Using the criteria described in the Materials and Methods, 53 potential MITEs were identified. The mean MITE length was 246 bp, the mean direct repeat length was 2.3 bp, and the mean terminal-inverted repeat length was 8.8 bp.

### BAC library construction and characterization

The bald cypress BAC library consists of 606,336 individually-archived clones in 1579 384-well microtiter plates. The library was given the designation TDD_Ba in accordance with the library naming standards used by the Mississippi Genome Exploration Laboratory (MGEL; see http://www.mgel.msstate.edu/dna_libs.htm), the Clemson University Genomics Institute (http://www.genome.clemson.edu), and the Arizona Genomics Institute (http://www2.genome.arizona.edu). The library and its associated products are distributed by MGEL (http://www.mgel.msstate.edu).

**Figure 3:**
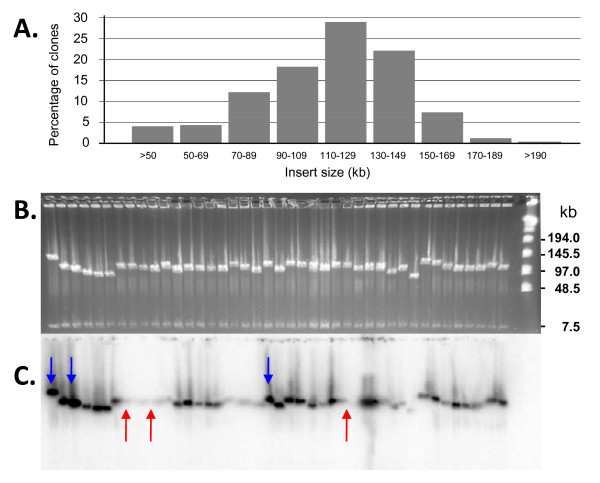
**Characterization of the bald cypress BAC library**. (A) Size distribution of BAC inserts in the TDD_Ba library as determined by pulsed-field gel electrophoresis (PFGE) of *Not*I-digested BAC clones. (B) Image of a PFGE gel showing *Not*I digested BAC clones. The BAC vector is visible as a 7.5 kb band near the bottom of each sample lane. The New England BioLabs Lambda Ladder is shown in the far right lane. (C) Southern blot of the gel shown in *B *hybridized with bald cypress genomic DNA. Note that some clones exhibit strong hybridization (blue arrows) while others exhibit relatively weak hybridization (red arrows). Clones with strong hybridization signals ostensibly contain more repetitive DNA than those with weak signals.

Periodically during BAC library construction, pulsed-field gel electrophoresis analysis was used to check the insert size of randomly-selected *Not*I-digested BACs. Inserts ranged in size from 10 kb to 202 kb (Figure 3A), and the mean insert size for the library was 113 kb (Figure 3B). The origin of the BAC insert DNAs was confirmed by Southern hybridization using radiolabeled bald cypress genomic DNA as a probe. As shown in Figure 3C, there is considerable variation in hybridization intensity between BAC inserts (Figure 3C) that cannot be accounted for by differences in DNA amount per lane (compare Figures 3B and 3C). Those clones with the strongest hybridization signals ostensibly contain more repetitive DNA than those with weaker signals.

Approximately 1.8% of clones in the library were false positives (i.e., they possessed a vector plasmid but had no discernible insert). The proportion of false positive clones was greater earlier in library construction - e.g., the first 660 plates had a false positive percentage of 3.05 while the remaining 919 plates had a false positive percentage of 0.95. The addition of a "pre-electrophoresis" size-selection step (see Materials & Methods) was likely responsible for the reduced number of false positives in the latter three-fifths of the library.

The level of chloroplast DNA contamination in the library was estimated by macroarray hybridization. Four loblolly pine (*Pinus taeda *L.) sequences representing different regions of the chloroplast genome [31] were used to probe five 4 × 4 double-spotted bald cypress macroarrays. We found that 2348 (approximately 2.5%) inserts of the 92,160 *Taxodium *clones represented on the macroarrays contained chloroplast DNA. This is the highest level of chloroplast contamination for any plant BAC library we have constructed, but still within the range of reported values (see reference [31] for discussion).

While macroarray screening with mitochondrial DNA was not performed, sequencing of nuclear DNA prepared using our nuclear DNA isolation protocol suggests that mitochondrial DNA contamination is 10 to 100 times less frequent than contamination from chloroplast DNA (reference 31 and Figure 2).

After subtracting the fractions of false positive clones and chloroplast DNA-containing clones, the number of clones containing bald cypress nuclear DNA was estimated to be 580,263. Thus the fraction of clones in the library containing nuclear DNA was 0.957 (i.e., 606,336 ÷ 580,263 = 0.957), and the number of nuclear DNA-containing clones on each macroarray was determined to be 17,639 (i.e., 18,432 × 0.957). Using the average insert size of 113 kb, the library contains approximately 6.74 genome equivalents of bald cypress nuclear DNA [(580,263 × 113 kb) ÷ 9731 Mb = 6.74)]. This level of genome equivalent coverage affords a 99.88% chance that a particular genomic sequence of interest will be found at least once in a library [see 39].

To further assess the quality and the utility of the library, eight *T. distichum *var. *distichum *single-copy gene sequences, employed previously in molecular phylogenetics research [40-42], were used to screen five 4 × 4 macroarrays. The five macroarrays collectively represented 1.02 (rounded to 1.0 for simplicity) genome equivalents of bald cypress nuclear DNA. The gene sequences (Table 2) were used to design PCR primers and/or overgo probes for each gene (Additional file 2, Tables S1 and S2), and macroarrays were separately screened with a pool containing the radiolabeled overgos and a pool containing the radiolabeled PCR amplicons. As shown in Table 3, the average number of positive clones recognized by a probe was 1.4 for the overgo pool and 1.5 for the amplicon pool. To verify the utility of the library in gene isolation, positive clones 1069I8, 1052C3 and 1137O7 were spotted onto nylon membranes and probed with various combinations of the overgo probes for the *Chi1*, *Ferr*, *Pat*, *HemA *and *Lcyb *genes. The results of these dot-blot hybridizations revealed that clones 1069I8, 1052C3, and 1137O7 contained the *Chi1*, *HemA*, and *Pat *genes (in full or part), respectively.

**Table 2:** Bald cypress genes used in macroarray analysis

Gene	Gene abbreviation	GenBank Accession
Putative ammonium transporter	*AMT*	AB211839
Aquaporin	*AQU*	AB211841
Calmodulin	*Cal*	AB211840
Pollen allergen	*Cryj2*	AB211842
Class I chitinase	*Chi1*	AB096607
Ferredoxin	*Ferr*	AB096608
Glutamyl-tRNA reductase	*HemA*	AB161815
Lycopene beta cyclase	*Lcyb*	AB096608
Phosphoribosylanthranilate transferase	*Pat*	AB161910

**Table 3:** Interrogation of macroarrays with overgo and PCR amplicon gene-specific probes

# of probes in hyb. mix	Genes represented by probes in hyb. mix	Probe type^a^	# of pos. clones^b^	Library addresses of positive clones^c^	Mean hits per genome
5	*Chi1*, *Ferr*, *Pat*, *HemA*, *Lcyb*	OVG	7	1052C3^d^, 1069I8^d^, 1137O7^d^, 1227H24, 1258G13, 1271L2, 1276E22	1.4
8	AMT, AQU, *Cal*, *Chi1*, *Cryj2*, *Ferr*, *Lcyb*, *HemA*	PCR	12	721G13, 724I23, 738H3, 749O14, 775F3, 813M13, 822N2, 826H4, 835L14, 836I14, 864F18, 885D20	1.5

^a^OVG = overgo (22-mer); PCR = PCR amplicon (each ca. 500 bp)

^b^Per one genome equivalent, i.e., per 73,728 clones

^c^Clone addresses are composed of a plate number, a row letter, and a column number, e.g., 1052N22 is the clone found in plate 1052, row N, column 22.

^d^A dot-blot experiment verified that clones 1052N22, 1069H17, and 1137O7 contained the *HemA*, *Chi1*, and *Pat *genes, respectively. Specific clone/probe relationships were not determined for the other clones.

### Library screening with repetitive sequences of bald cypress

For estimation of repetitive DNA content, blots prepared from pulsed-field gels of random *NotI*-digested BAC clones were probed with radiolabeled Cot ≤ 1 M·s (i.e., Cot-1) DNA. Based on the Cot data, Cot-1 DNA should contain sequences repeated, on average, 9523 times. Among the 328 clones on the various Southern blots, 273 showed obvious hybridization signals using the Cot-1 probe, i.e., approximately 83.2% of the BAC clones in the library appear to have inserts containing repetitive elements found hundreds to thousands of times in the genome (data not shown).

**Figure 4:**
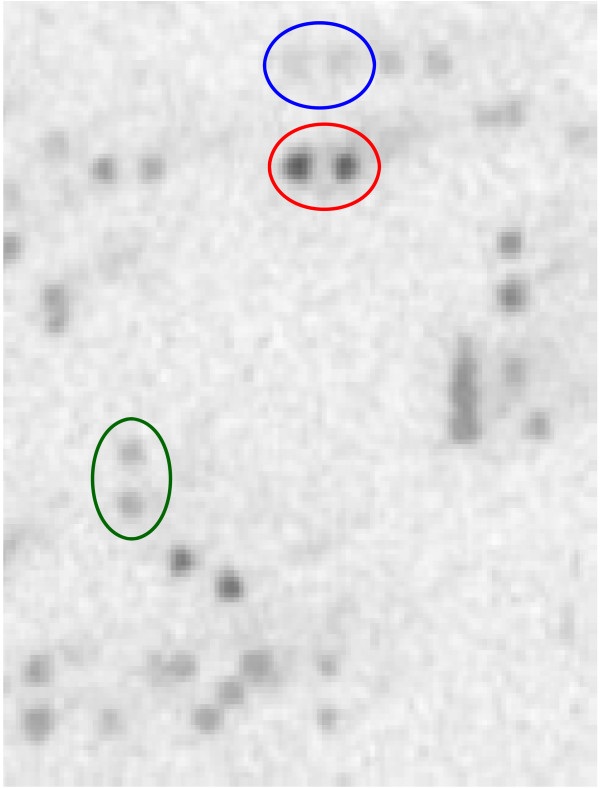
**TdCRT1 hybridization to BAC clones**. Close-up of a portion of a bald cypress 4 × 4 macroarray probed with the TdCRT1 overgo. Because each clone is double-spotted on the macroarray, positive signals are indicated by closely adjoined dots of similar hybridization intensity (e.g., colored circles). Presumably clones exhibiting the most intense hybridization (e.g., clone in red circle) contain the highest number of TdCRT1 sequences while clones with intermediate or low numbers of TdCRT1 exhibit moderate and low hybridization, respectively (e.g., clones in green and blue circles).

To study the distribution of a particular retroelement in the *Taxodium *genome, a 4 × 4 macroarray was screened with an overgo probe (Additional file 2, Table S3) designed from a bald cypress HR Cot-filtered sequence [GenBank: ET185333] with high sequence identity (S' = 128) to two *Gingko biloba copia-*like retroelement reverse transcriptase sequences [GenBank: DQ054445, GenBank: DQ054446]. Of the 17,639 nuclear DNA-containing clones on the macroarray, 888 (i.e., 10.1%) exhibited hybridization to the overgo which represents a reverse transcriptase gene we have deemed TdCRT1 for *Taxodium distichum copia*-like reverse transcriptase 1 (Figure 4). Densitometer analysis of the macroarray suggests that TdCRT1 is found in roughly 23,892 copies in the bald cypress genome. An initial glance at TdCRT1 hybridization to macroarrays suggests that the element is found in clusters, i.e., it is not distributed randomly throughout the genome. To test this hypothesis, we used the probabilistic "urn model" method of Holst [43] as detailed in Shan et al. [44]. Using an average BAC insert size of 113 kb, an estimated 17,639 nuclear DNA-containing clones per macroarray, and a genome size of 9731 Mb, we calculated that each macroarray represents about 0.205X coverage of the *Taxodium *genome [i.e., 17,639 · 113 kb)/9731 Mb = 0.205]. Thus one would predict that one macroarray should contain 4,898 copies of TdCRT1 (i.e., 23,892 copies · 0.205 = 4,898). If TdCRT1 distribution were indeed random, we would expect that the distribution of clones lacking TdCRT1 (i.e., lacking hybridization signal) would approximate normality. The mean number of clones expected to lack a TdCRT1-containing element and the theoretical standard deviation (*SD*) can be estimated using Theorem 2 of Holst [43]. Specifically,

$$mean=Ne^{-np_{k}}\text{ ; }SD={\left(\frac{n^{2}Np_{k}^{2}}{2}\right)}^{0.5}$$

where *N *is the number of nuclear DNA-containing clones on the macroarray (i.e., 17,639), *n *is the expected number of clones showing TdCRT1 hybridization (i.e., 4,898), and *p_k _*is the probability of any copy of the element "falling" within a clone (i.e., 1/17,639). Insertion of the preceding values into the equations produced a mean of 13,362 clones per macroarray with a standard deviation of 26. However, we determined that 16,751 of the 17,639 clones on the macroarray did not exhibit TdCRT1 hybridization. Consequently, the observed number of clones lacking TdCRT1 hybridization is 642 times (16,751/26 = 642) the expected standard deviation for a normal distribution, strongly supporting the premise that the distribution of TdCRT1 is not random.

Of note, nine overgos representing common *Ty1-copia*-like or *Ty3-gypsy*-like repeats from loblolly pine were hybridized to bald cypress macroarrays. None of the loblolly pine repeat-based overgos hybridized to the *Taxodium *filters (data not shown).

## Discussion

The genome of bald cypress, a Cupressaceae conifer, is three times the size of the human genome but only half the size of the genomes of most Pinaceae conifers [12]. Bald cypress appears to be a diploid like its Pinaceae cousins and, since evidence suggests that conifers do not likely require or utilize more genes than other seed plants [45], a natural hypothesis is that the bald cypress genome contains lots of repetitive, non-coding DNA. Our Cot analysis supports this general tenet as the repetitive fraction of the bald cypress genome is substantial (HR + MR = 90.5%). However, the HR and MR components of bald cypress have relatively low mean repetition frequencies (2054 and 61, respectively). The idea that bald cypress repeats tend to be found in low copy numbers is supported by analysis of the HR sequences with the *de novo *repeat detection functions of the Sequence Read Classification Pipeline (SRCP) [36]. SRCP analysis reveals that only 6.2% of 743 bald cypress HR reads showed enough sequence identity with each other to be grouped by the SRCP into the "Probable Repeats" category. In comparison, SRCP evaluation of random Sanger sequence reads from *Sorghum bicolor *(sorghum), a plant with a genome one-thirteenth the size of bald cypress [46], resulted in categorization of 27% of *Sorghum *reads as "Probable Repeats" despite the fact that the *Sorghum *sequences were not specifically enriched for repeats [36].

Why the *Taxodium *genome has so much low-copy repetitive DNA is unknown. A reasonable guess is that the majority of mobile element amplification events in bald cypress occurred tens to hundreds of millions of years ago, and since then element proliferation/activity has been severely restricted. If mobile element amplification was suppressed long ago, the copies of each repetitive element would presumably begin to diverge until they were eventually no longer readily recognizable as being derived from the same source. Indeed, the limited studies of mobile elements in conifers do indicate that there is considerable sequence divergence among members of retroelement families [31,47,48]; moreover, much of the DNA within sequenced regions of conifer genomes lacks features characteristic of known mobile elements, low-complexity repeats, or genes [31,49]. Since all conifers possess enormous genomes, it is possible that some of the mobile element amplification events that underlie the behemoth C-values of conifers occurred prior to the divergence of the major conifer families; this idea should be much easier to test once genome sequences become available for conifers.

Only 0.54% of reads (i.e., four reads total) showed reasonable homology (S' = 60) to previously annotated transposons; top hits were to *copia*-like retroelements from *Ginkgo biloba *(GenBank: DQ054445, GenBank: DQ054446), *Sequoiadendron giganteum *(AJ290723), and *Picea glauca *[GenBank: AF229252], and a *gypsy*-like element from *Abies veitchii *[GenBank: AJ002621]. The HR read with the highest level of similarity to a previously characterized retroelement [GenBank: ET185333] was used to design an overgo probe (see Additional file 2, Table S3) that was hybridized to bald cypress macroarrays. The element represented by the overgo, i.e., TdCRT1, was estimated to be present in approximately 23,892 copies per 1C genome. As described above, 80% of HR sequences are present in copy numbers between 205.4 and 20,540. If half the remaining 20% of sequences in the HR component reassociate after 10·Cot1/2, then we would predict that the remaining half (i.e., 10%) would reassociate prior to 0.1·Cot1/2. In other words, we would expect less than 10% of HR sequences to have an iteration frequency greater than 20,540. Since TdCRT1 has a frequency of 23,892 copies per genome, it seems reasonable to assume that few, if any, genome sequences are more redundant than TdCRT1. Statistical evaluation of the distribution of TdCRT1 among clones on a macroarray suggests that it is found in a decidedly non-random distribution throughout the bald cypress genome. That one of the most redundant sequences in the 9.7 Gb *Taxodium *genome is found in only 23,892 copies and is distributed in a decidedly non-random fashion initially struck us as a bit surprising. However, as Baucom et al. [50] note, "Two of the many misconceptions about TE (transposable element) properties in higher eukaryotes are that they are highly repetitive and are randomly scattered about the genome."

Our previous studies on the loblolly pine genome suggest that, as in bald cypress, the majority of pine repetitive DNA sequences are highly diverged and ostensibly ancient. However, in contrast to bald cypress, there appear to be at least a few retroelement families in loblolly pine of more pronounced conservation (and ostensibly of more recent origin). For example, the retroelement *IFG7 *[51] is found in 210,557 copies and accounts for about 5.8% of the pine genome [31] while the *Athila*-like retroelement *Gymny *is found in approximately 21,700 copies and accounts for 1.3% of the loblolly pine genome [47]. Together, *IFG7 *and *Gymny *make up 1.54 Gb of the loblolly pine genome, a value 9.8 times that of the *Arabidopsis thaliana *genome [52]. As the pine genome is roughly twice the size of that of bald cypress, it is possible that the activity of *IFG-7*, *Gymny*, and other mobile element families are, in part, responsible for the larger genome of loblolly pine (and other Pinaceae conifers) compared to bald cypress.

The SL component of bald cypress accounts for 7.4% of its genome. This is an amount 4.6 times the size of the *Arabidopsis *genome. Because conifers appear to have functional gene numbers similar to those of diploid angiosperms [45], it is possible that much of the SL DNA of *Taxodium *is composed of repetitive elements that have diverged to the point that they are now "single-copy" in nature, and indeed, studies on other conifers have suggested that the high sequence complexity of their low-copy DNA is likely due, in part, to repeat divergence [31,47,48]. Duplication of genes and/or pseudogene production may also contribute to the size of the SL component. It is interesting to note that the macroarray hybridization results using probes for *Taxodium *"single-copy" genes (Table 2) indicate that there are 1.4-1.5 positive clone hits per probe per estimated 1C genome equivalent. This result could indicate that BAC library coverage is actually greater than what we calculated based on insert size and clone number or that the genome size of bald cypress is actually smaller than previously reported. Alternatively, some of our probes may be hybridizing to multiple loci. Because our analysis was based upon macroarray hybridization and not PCR, it is also possible that some of the amplicon and/or overgo probes we developed contain regions with significant sequence identity to loci not amplified using the primers from the previous phylogenetic studies. Such loci could be paralogous genes or pseudogenes. Of note, pseudogenes have been described in a number of Pinaceae conifers including pines [49,53-56], larches [57], and spruces [58,59]. In a recent examination of ten BAC sequences from loblolly pine, Kovach et al. [49] found that pseudogenes were five times more common than genes with potential protein coding functions - whether such a high pseudogene level holds for the genome as a whole is unknown. To our knowledge, pseudogenes have not been reported in Cupressaceae conifers, although the amount of sequence information for Cupressaceae is minute compared to that for Pinaceae. As a means of exploring whether pseudogenes are common in bald cypress, we plan to sequence *Taxodium *BAC clones including those recognized by the probes listed in Table 2.

Hybridization of loblolly pine retroelement sequence probes to macroarrays of *Taxodium *produced no positive signals. This is not particularly surprising as fossil evidence indicates that the Pinaceae and the Cupressaceae have been separate lineages for roughly 250 million years [1].

Analysis of the bald cypress HR sequence with MUST [37] resulted in detection of 53 potential MITEs (Additional file 1). None of the MITEs shared terminal-inverted repeats, and hence each potential MITE was considered a member of its own MITE family. Further analysis of the potential MITEs will be facilitated by sequencing of bald cypress BACs and random genomic DNA.

## Conclusions

We have explored the genome of bald cypress using several molecular techniques. Of particular note, we have generated a BAC library for bald cypress. This is the first large-insert library for a member of the Cupressaceae and a logical tool in eventual sequencing and assembly of the bald cypress genome. With regard to genome biology, the nuclear DNA of bald cypress appears to be largely composed of relatively low-copy repeats. These low-copy sequences may have arisen from ancient mobile element amplification events followed by millions of years of mobile element quiescence. Sequencing of BACs should provide key information on gene structure while helping to define the roles that gene duplication, pseudogenes, and repeat sequence divergence have played in shaping the *Taxodium *genome.

## Methods

### Plant materials and DNA isolation

Young leaves were collected from a bald cypress tree located at 33° 27.2729' N, 88° 47.5898' W on the Mississippi State University campus, and nuclear DNA was isolated according to the protocol described in Additional file 3. The DNA was sonicated into fragments with a mean length of 450 bp (Additional file 4), and metal ions were removed from the DNA solution using Chelex-100 (Additional file 5). The fragmented DNA was ethanol precipitated and re-dissolved in 0.5 M sodium phosphate buffer (Additional file 6) for Cot analysis or 10 mM Tris-HCl (pH 8.0) for other uses.

### Melting curve and Cot analysis

DNA preparation and melting analyses were performed as described previously [60]. Cot analysis was performed according to Peterson et al. [20,60]. Cot data was analyzed using the program CotQuest [34].

### Cot filtration and highly repetitive DNA library construction

From the Cot analysis we determined that the Cot1/2 value of the HR component was 4.64 M·s (Table 1). To isolate the HR component and foldback DNA, a bald cypress DNA sample was denatured and allowed to reassociate to Cot 6 M·s (rounded up from 4.64 M·s for simplicity). The resulting double-stranded DNA (Cot ≤ 6 M·s) was isolated using hydroxyapatite chromatography and ligated into either the pGEM T-Easy (Promega) or TOPO TA (Invitrogen) cloning vector. The resulting recombinant molecules were used to transform ElectroMAX DH10B T1 Phage-Resistant *E. coli *cells (Invitrogen) according to Peterson et al. [20]. HR clones were end-sequenced (single-pass) using an ABI 3730×l DNA analyzer. We obtained high-quality reads for 743 clones. The HR clone sequences were deposited in GenBank (GenBank: ET185231-ET185973).

### HR sequence analysis

The HR sequence reads were analyzed using the Sequence Read Classification Pipeline (SRCP) previously described in Chouvarine et al. [36]. Additionally, the sequences were evaluated using the miniature inverted-repeat transposable element (MITE) identification tool MUST [37] with the following parameters: minimum TIR length = 8 nt; maximum TIR length = 50 nt; minimum DR length = 2 nt; maximum DR length = 30 nt; minimum MITE length = 100 nt; maximum MITE length = 1000 nt; minimum in-cluster identity = 1. In Figure 2, potential MITEs detected by MUST were only included under the MITE heading if they were categorized as either "Unknown" or "Probable Repeat" by the SRCP. For a putative MITE in an SRCP-classified "Unknown" or "Probable Repeat" read, only the length of the MITE itself (TIR and inter-TIR sequence) was considered MITE sequence; the remainder of the bases in the read were classified based upon the read's SRCP categorization.

### BAC library construction and characterization

Young leaves were obtained from a bald cypress tree located at 33° 27.4330' N, 88° 47.3777' W on the Mississippi State University campus. We have designated this tree MSSTATE#1, and rooted cuttings from the tree have been sent to the USDA's Southern Institute for Forest Genetics (Saucier, MS) for maintenance and distribution. A BAC library was produced from MSSTATE#1 leaf nuclear DNA according to Peterson et al. [61]. A "pre-electrophoresis" step, as detailed in Magbanua et al. [31], was added to the BAC library protocol during the size-selections preceding the production of plates 661-919. Introduction of the "pre-electrophoresis" step appears to have led to a decrease in false positives clones in the latter 60% of the library compared to the first 40% (see Results).

Preparation of radiolabeled probes and macroarray hybridization were conducted as described in Magbanua et al. [31]. Southern hybridization was conducted using standard techniques [62].

### Assessment of repeats in BAC library

The macroarray image showing TdCRT1 hybridization was analyzed using an AlphaInnotech densitometer. Repeat copy numbers were estimated from macroarrays as described in Magbanua et al. [31].

## Authors' contributions

WL constructed the BAC library and designed and performed macroarray hybridization experiments. ST generated the Cot curve and performed Cot-filtration. WL and ST put together the first draft of the manuscript. SKS performed data analysis and helped refine the text. PC participated in sequence and Cot curve analysis. DGP conceived the project, participated in sequence and Cot analysis, and conducted extensive manuscript editing. All authors read and approved the manuscript.

## Supplementary Material

Additional file 1 **Miniature inverted-repeat transposable elements detected in bald cypress HR sequences**. Cot-filtered, highly repetitive (HR) sequences containing MITEs are listed in FASTA format. Each MITE is represented by a gold highlighted internal sequence flanked on each end by a terminal inverted-repeat sequence (green highlight) and a target site duplication (direct repeat, yellow highlight). Click here for file

Additional file 2 **Primers and overgos used in macroarray analysis**. Click here for file

Additional file 3 **Isolation of nuclear DNA from plants**. This is the standard Mississippi Genome Exploration Laboratory (MGEL) nuclear DNA isolation protocol used to obtain the milligram quantities of plant nuclear DNA required for Cot analysis and Cot filtration. Click here for file

Additional file 4 **Shearing DNA into 450 bp fragments using the Misonix Sonicator 3000**. This is a detailed MGEL protocol describing the method used to shear *Taxodium distichum *nuclear DNA for use in Cot analysis and Cot filtration. Click here for file

Additional file 5 **Removing metal ions from DNA solutions using Chelex**. This is a detailed MGEL protocol describing removal of metal ions from DNA solutions using Chelex. Click here for file

Additional file 6 **Preparing 0.5 M sodium phosphate buffer (SPB)**. This is a detailed MGEL protocol describing how to prepare sodium phosphate buffer for use in Cot analysis and Cot filtration. Click here for file

## References

[B1] Farjon A A Natural History of Conifers 2008 Portland, OR: Timber Press

[B2] Brown CA Montz GN Baldcypress: The tree unique, the wood eternal 1986 Baton Rouge, LA: Claitor's Publishing Division

[B3] Burns RM Honkala BH (Eds) Silvics of North America. Volume 1: Conifers Agric Handbk 654 1990 Washington, D.C.: U.S. Department of Agriculture Forest Service

[B4] Parkinson J Theatrum Botanicum London: Thomas Coates 1640

[B5] Brown P Dean JS, Meko DM, Swetnam TW OLDLIST: A database of maximum tree ages Tree Rings, Environment, and Humanity 1996 Tucson, AZ: Radiocarbon (Univ. of Arizona) 727 731

[B6] Wilhite LP Toliver JR Burns RM, Honkala BH *Taxodium distichum *(L.) Rich., Baldcypress Silvics of North America. Volume 1, Conifers Agric Handbk 654 1990 Washington, D.C.: U.S. Department of Agriculture Forest Service 563 572

[B7] Watson FD The nomenclature of pondcypress and bald cypress (Taxodiaceae) Taxonomy 1986 34 506 509

[B8] Tsumura Y Tomaru N Suyama Y Bacchus S Genetic diversity and differentiation of *Taxodium *in the south-eastern United States using cleaved amplified polymorphic sequences Heredity 1999 83 Pt 3 229 238 10504419 10.1038/sj.hdy.6885810

[B9] Lickey EB Walker GL Population genetic structure of baldcypress (*Taxodium distichum *(L.) Rich. *var. distichum*) and pondcypress (*T. distichum var. imbricarium *[Nuttall] Croom): Biogeographic and taxonomic implications Southeastern Naturalist 2002 1 2 131 148 10.1656/1528-7092(2002)001[0131:PGSOBT]2.0.CO;2

[B10] Debreczy Z Rácz I El Arbol del Tule: The ancient giant of Oaxaca Arnoldia: The Magazine of the Arnold Arboretum 1997 57 4 3 11

[B11] Hizume M Kondo T Shibata F Ishizuka R Flow cytometric determination of genome size in the Taxodiaceae, Cupressaceae *sensu stricto *and Sciadopityaceae Cytologia 2001 66 307 311 10.1508/cytologia.66.307

[B12] Murray BG Leitch IJ Bennett MD Gymnosperm DNA C-values database (release 3.0, Dec. 2004) 2004 http://www.rbgkew.org.uk/cval/homepage.html

[B13] Schlarbaum SE Johnson LC Tsuchiya T Chromosome studies of *Metasequoia glyptostroboides *and *Taxodium distichum* Botanical Gazette 1983 144 4 559 565 10.1086/337411

[B14] Ujino-Ihara T Yoshimura K Ugawa Y Yoshimaru H Nagasaka K Tsumura Y Expression analysis of ESTs derived from the inner bark of *Cryptomeria japonica* Plant Mol Biol 2000 43 4 451 457 10.1023/A:1006492103063 11052197

[B15] Tani N Takahashi T Iwata H Mukai Y Ujino-Ihara T Matsumoto A Yoshimura K Yoshimaru H Murai M Nagasaka K Tsumura Y A consensus linkage map for sugi (*Cryptomeria japonica*) from two pedigrees, based on microsatellites and expressed sequence tags Genetics 2003 165 1551 1568 14668402 10.1093/genetics/165.3.1551 PMC1462850

[B16] Mukai Y Suyama Y Tsumura Y Kawahara T Yoshimaru H A linkage map for sugi (*Cryptomeria japonica*) based on RFLP, RAPD and isozyme loci Theor Appl Genet 1995 90 835 840 10.1007/BF00222019 24172926

[B17] Britten RJ Kohne DE Repeated sequences in DNA Science 1968 161 529 540 10.1126/science.161.3841.529 4874239

[B18] Britten RJ Graham DE Neufeld BR Analysis of repeating DNA sequences by reassociation Methods Enzymol 1974 29 363 418 4850571 10.1016/0076-6879(74)29033-5

[B19] Peterson DG Meksem K, Kahl G Reduced representation strategies and their application to plant genomes The Handbook of Genome Mapping: Genetic and Physical Mapping 2005 Weinheim: WILEY-VCH Verlag GmbH & Co. KGaA 307 335

[B20] Peterson DG Schulze SR Sciara EB Lee SA Bowers JE Nagel A Jiang N Tibbitts DC Wessler SR Paterson AH Integration of Cot analysis, DNA cloning, and high-throughput sequencing facilitates genome characterization and gene discovery Genome Res 2002 12 795 807 10.1101/gr.226102 11997346 PMC186575

[B21] Peterson DG Wessler SR Paterson AH Efficient capture of unique sequences from eukaryotic genomes Trends Genet 2002 18 11 547 550 10.1016/S0168-9525(02)02764-6 12414178

[B22] Paterson AH Leafing through the genomes of our major crop plants: strategies for capturing unique information Nat Rev Genet 2006 7 3 174 184 16485017 10.1038/nrg1806

[B23] Yuan Y SanMiguel PJ Bennetzen JL High-Cot sequence analysis of the maize genome Plant J 2003 34 2 249 255 10.1046/j.1365-313X.2003.01716.x 12694599

[B24] Lamoureux D Peterson DG Li W Fellers JP Gill BS The efficacy of Cot-based gene enrichment in wheat (*Triticum aestivum *L.) Genome 2005 48 6 1120 1126 10.1139/g05-080 16391681

[B25] Wicker T Robertson JS Schulze SR Feltus FA Magrini V Morrison JA Mardis ER Wilson RK Peterson DG Paterson AH Ivarie R The repetitive landscape of the chicken genome Genome Res 2005 15 126 136 10.1101/gr.2438005 15256510 PMC540276

[B26] Shizuya H Birren B Kim U-J Mancino V Slepak T Tachiiri Y Simon M Cloning and stable maintenance of 300-kilobase-pair fragments of human DNA in *Escherichia coli *using an F-factor-based vector Proc Natl Acad Sci USA 1992 89 8794 8797 10.1073/pnas.89.18.8794 1528894 PMC50007

[B27] Zhang H-B Wu C BACs as tools for genome sequencing Plant Physiol Biochem 2001 39 195 209 10.1016/S0981-9428(00)01236-5

[B28] Cai W-W Reneker J Chow C-W Vaishnav M Bradley A An anchored framework BAC map of mouse chromosome 11 assembled using multiplex oligonucleotide hybridization Genomics 1998 54 387 397 10.1006/geno.1998.5620 9878241

[B29] Chen M Presting G Barbazuk WB Goicoechea JL Blackmon B Fang G An integrated physical and genetic map of the rice genome Plant Cell 2002 14 537 545 10.1105/tpc.010485 11910002 PMC150577

[B30] Lin L Pierce GJ Bowers JE Estill JC Compton RO Rainville LK A draft physical map of a D-genome cotton species (*Gossypium raimondii*) BMC Genomics 2010 11 395 10.1186/1471-2164-11-395 20569427 PMC2996926

[B31] Magbanua ZV Ozkan S Bartlett BD Chouvarine P Saski CA Liston A Cronn RC Nelson CD Peterson DG Adventures in the enormous: A 1.8 million clone BAC library for the 21.7 Gb genome of loblolly pine PLoS ONE 2011 6 1 e16214 10.1371/journal.pone.0016214 21283709 PMC3025025

[B32] Bautista R Villalobos DP Díaz-Moreno S Canton FR Canovas FM Claros MG Toward a *Pinus pinaster *bacterial artificial chromosome library Ann Forest Sci 2007 64 8 855 864 10.1051/forest:2007060

[B33] Hamberger B Hall D Yuen M Oddy C Hamberger B Keeling CI Ritland C Ritland K Bohlmann J Targeted isolation, sequence assembly and characterization of two white spruce (*Picea glauca*) BAC clones for terpenoid synthase and cytochrome P450 genes involved in conifer defence reveal insights into a conifer genome BMC Plant Biol 2009 9 106 10.1186/1471-2229-9-106 19656416 PMC2729077

[B34] Bunge J Chouvarine P Peterson DG CotQuest: Improved algorithm and software for nonlinear regression analysis of DNA reassociation kinetics data Anal Biochem 2009 388 322 330 10.1016/j.ab.2009.03.007 19285476

[B35] Hood LE Wilson JH Wood WB Molecular Biology of Eucaryotic Cells 1975 Menlo Park, CA: W.A. Benjamin

[B36] Chouvarine P Saha S Peterson DG An automated, high-throughput sequence read classification pipeline for preliminary genome characterization Anal Biochem 2008 373 78 87 10.1016/j.ab.2007.08.008 17868636

[B37] Chen Y Zhou F Li G Xu Y MUST: a system for identification of miniature inverted-repeat transposable elements and applications to *Anabaena variabilis *and *Haloquadratum walsbyi* Gene 2009 436 1-2 1 7 10.1016/j.gene.2009.01.019 19393167

[B38] Kuang H Padmanabhan C Li F Kamei A Bhaskar PB Ouyang S Jiang J Buell CR Baker B Identification of miniature inverted-repeat transposable elements (MITEs) and biogenesis of their siRNAs in the Solanaceae: new functional implications for MITEs Genome Res 2009 19 1 42 56 19037014 10.1101/gr.078196.108 PMC2612961

[B39] Plomion C Chagné D Pot D Kumar S Wilcox PL Burdon RD Prat D Peterson DG Paiva J Chaumeil P Vendramin GG Sebastiani F Nelson CD Echt CS Savolainen O Kubisiak TL Cervera MT de María N Islam-Faridi MN Kole CR The Pines Genome Mapping and Molecular Breeding in Plants, Vol. 7 - Forest Trees 2007 Heidelberg, Berlin, New York, Tokyo: Springer 29 78

[B40] Kusumi J Tsumura Y Yoshimaru H Tachida H Molecular evolution of nuclear genes in Cupressacea, a group of conifer trees Mol Biol Evol 2002 19 736 747 11961107 10.1093/oxfordjournals.molbev.a004132

[B41] Kado T Ushio Y Yoshimaru H Tsumura Y Tachida H Contrasting patterns of DNA variation in natural populations of two related conifers, *Cryptomeria japonica *and *Taxodium distichum *(Cupressaceae *sensu lato*) Genes Genet Syst 2006 81 2 103 113 10.1266/ggs.81.103 16755134

[B42] Kusumi J Zidong L Kado T Tsumura Y Middleton BA Tachida H Multilocus patterns of nucleotide polymorphism and demographic change in *Taxodium distichum *(Cupressaceae) in the lower Mississippi River alluvial valley Am J Bot 2010 97 11 1848 1857 10.3732/ajb.1000082 21616823

[B43] Holst L Limit Theorems for Some Occupancy and Sequential Occupancy Problems Ann Mathemat Stat 1971 42 5 1671 1680 10.1214/aoms/1177693165

[B44] Shan X Ray DA Bunge JA Peterson DG A bacterial artificial chromosome library for the Australian saltwater crocodile (*Crocodylus porosus*) and its utilization in gene isolation and genome characterization BMC Genomics 2009 10 Suppl 2 S9 10.1186/1471-2164-10-S2-S9 19607660 PMC2966330

[B45] Rigault P Boyle B Lepage P Cooke JE Bousquet J MacKay JJ A white spruce gene catalogue for conifer genome analyses Plant Physiol 2011 157 1 14 28 10.1104/pp.111.179663 21730200 PMC3165865

[B46] Paterson AH Bowers JE Bruggmann R Dubchak I Grimwood J Gundlach H The *Sorghum bicolor *genome and the diversification of grasses Nature 2009 457 7229 551 556 10.1038/nature07723 19189423

[B47] Morse AM Peterson DG Islam-Faridi MN Smith KE Magbanua Z Garcia SA Kubisiak TL Amerson HV Carlson JE Nelson CD Davis JM Evolution of genome size and complexity in *Pinus* PLoS ONE 2009 4 e4332 10.1371/journal.pone.0004332 19194510 PMC2633040

[B48] Elsik CG Williams CG Retroelements contribute to the excess low-copy-number DNA in pine Mol Gen Genet 2000 264 47 55 10.1007/s004380000279 11016832

[B49] Kovach A Wegrzyn JL Parra G Holt C Bruening GE Loopstra CA Hartigan J Yandell M Langley CH Korf I Neale DB The *Pinus taeda *genome is characterized by diverse and highly diverged repetitive sequences BMC Genomics 2010 11 420 10.1186/1471-2164-11-420 20609256 PMC2996948

[B50] Baucom RS Estill JC Chaparro C Upshaw N Jogi A Deragon JM Westerman RP SanMiguel PJ Bennetzen JL Exceptional diversity, non-random distribution, and rapid evolution of retroelements in the B73 maize genome PLoS Genet 2009 5 11 e1000732 10.1371/journal.pgen.1000732 19936065 PMC2774510

[B51] Kossack DS Kinlaw CS IFG, a gypsy-like retrotransposon in *Pinus *(Pinaceae), has an extensive history in pines Plant Mol Biol 1999 39 3 417 426 10.1023/A:1006115732620 10092171

[B52] Bennett MD Leitch IJ Angiosperm DNA C-values database (release 5.0, Dec. 2004) 2004 http://www.kew.org/cvalues/homepage.html

[B53] Garcia-Gil MR Evolutionary aspects of functional and pseudogene members of the phytochrome gene family in Scots pine J Mol Evol 2008 67 2 222 232 10.1007/s00239-008-9135-z 18663508

[B54] Skinner JS Timko MP Loblolly pine (*Pinus taeda *L.) contains multiple expressed genes encoding light-dependent NADPH:protochlorophyllide oxidoreductase (POR) Plant Cell Physiol 1998 39 8 795 806 9787456 10.1093/oxfordjournals.pcp.a029437

[B55] Wakasugi T Tsudzuki J Ito S Nakashima K Tsudzuki T Sugiura M Loss of all ndh genes as determined by sequencing the entire chloroplast genome of the black pine *Pinus thunbergii* Proc Natl Acad Sci USA 1994 91 21 9794 9798 10.1073/pnas.91.21.9794 7937893 PMC44903

[B56] Gernandt DS Liston A Pinero D Variation in the nrDNA ITS of *Pinus *subsection Cembroides: implications for molecular systematic studies of pine species complexes Mol Phylogenet Evol 2001 21 3 449 467 10.1006/mpev.2001.1026 11741386

[B57] Wei XX Wang XQ Recolonization and radiation in *Larix *(Pinaceae): evidence from nuclear ribosomal DNA paralogues Mol Ecol 2004 13 10 3115 3123 10.1111/j.1365-294X.2004.02299.x 15367124

[B58] Kvarnheden A Tandre K Engstrom P A cdc2 homologue and closely related processed retropseudogenes from Norway spruce Plant Mol Biol 1995 27 2 391 403 10.1007/BF00020192 7888627

[B59] Kvarnheden A Albert VA Engstrom P Molecular evolution of cdc2 pseudogenes in spruce (*Picea*) Plant Mol Biol 1998 36 5 767 774 10.1023/A:1005901413475 9526509

[B60] Peterson DG Pearson WR Stack SM Characterization of the tomato (*Lycopersicon esculentum*) genome using *in vitro *and *in situ *DNA reassociation Genome 1998 41 346 356

[B61] Peterson DG Tomkins JP Frisch DA Wing RA Paterson AH Construction of plant bacterial artificial chromosome (BAC) libraries: An illustrated Guide J Agric Genomics 2000 5 http://wheat.pw.usda.gov/jag/

[B62] Sambrook J Fritsch EF Maniatis T Molecular Cloning: A Laboratory Manual 1989 Cold Spring Harbor, New York: Cold Spring Harbor Press

[B63] Britten RJ Davidson EH Hames BD, Higgins SJ Hybridisation strategy Nucleic Acid Hybridisation 1985 Washington, DC: IRL Press 3 15

