# Supplementary material for: Characterization of the genome of bald cypress
Source: BMC Genomics. 2011 Nov 11;12:553. doi: 10.1186/1471-2164-12-553 (PMC3228858; doi:10.1186/1471-2164-12-553)
Supplement: Additional file 1 — Miniature inverted-repeat transposable elements detected in bald cypress HR sequences. Cot-filtered, highly repetitive (HR) sequences containing MITEs are listed in FASTA format. Each MITE is represented by a gold highlighted internal sequence flanked on each end by a terminal inverted-repeat sequence (green highlight) and a target site duplication (direct repeat, yellow highlight). [file 1471-2164-12-553-S1.PDF]

Miniature inverted-repeat transposable elements detected in bald cypress HR sequences

Target-site duplications (direct repeats) are highlighted in yellow.
Terminal inverted-repeats are highlighted in green.
Internal non-coding sequences are highlighted in gold.

>gi|162316918|gb|ET185972.1
TTACCTCATTTGCACTATTGGACATTTTTTATAGCGTTGTGGCAAGGGTTGAGTGGTGCTTAAGACTAGAGGACCTC
AACCTATTCACTAATATAGAAATTTGGGCACCATTTTGTGTAAGGCGATTGATGAGGACCTCTTGCATGGCATCTAC
ATGATCTAATGGAGGACGTGGAGGGTATCGAGGTGGTGGCGGTTGTTGAGGAGAAACCTTTTGAGGATTTTGCA
ATTGTGGAGCAATGTCTTGAGGGTTTTCTTGTTGCCCATGTACAACGCTTATAGGGTTTGCTTGTGTTTCTTGTGCA
AGAGGAGGTCTTTGTTGTCTATTTCATTTTTGGGGATTTCTTGAAGAATCTAACATCAAATTAATAAAAAAAACTTA
AATCAATTAA
>gi|162316914|gb|ET185968.1
TTAGAAGGAACCTGGGCCGTATTACGTATAGAATCCAAATCGATCGATCTTGTATTGCTAGAGATCGATCGCTTTA
TCTTCCACCCACCCTACACTATTGCTTCCCACCCACCCGCATAGTTGTGATGAGATCGATCTTGAGCACTCGATTCTA
GCCACTATAATTGCTTAATCTAGCAGGCGTATTAATTGACCGGACTATAGTGACTGATTCGATTAAGTGGGAGGGC
AACTATCGAGTCCCCTCCCCACGTCATTTCTATAGTTGCTGATTCGATTAGTCGATTCTAGCACTCGATTAATGAACT
ATTATAGCTATAGTGACTGATTCGATTAGAGGAAGCTAAGGGGCCGACTAGCGAGTCCCTCCCACTTAATAACTAT
GCCCCCA
>gi|162316902|gb|ET185956.1
TAATTATTTTTTGAAACTAAGCCAAGAAAGATTATGTGACCCTGAGGGTCACATCAATAATGTTAAGAGAAAAATA
TTGACTAGCATAGGGGTCACTAATGAGGTGCTCTTAGATGAAGATGTGATTAGAAATTGTGTCACTTTTGATTAAA
TTTTTCAAAGAAGGAGAAAATGGAACATTTTGATAAAGATAGAGGAAGAGAAAAGAAAGAGGGATCCAAGATTC
CCTATATTTTAAATCGAAGAACCCAACATCCTTAAAAGGATTTATTTGCATATAAATAATTTTGAAAAATTGTATGG
TAAATAAGTTGGTGGTGAAACTTCACCAAAGAAGGCAAAATTGCTTGTAAAGAAACCCAAGGGGGTTGTGATTTG
TAATGATCCTAATGCCATATCTTTTCAAGAATCTTCAACCAATCTACTCCCACTGAAAAGACAAAAAAAGAAGATCA
TGTGCCCTTGGTTCCATATGATGACACTAAAGAAGTTGGTGACACACATGAGGTTGTTTATAGTGCTTTAAGGGCT
TTGAAAGATGATGAGGGAGTTGATCAACCCCATGGATCACCCCCTAGGTATAAAGAGGAAGAACATGGGCATGA
ATTTAGGGCTAAGGGGGAGACGTTGCTAGAAGATCAA
>gi|162316892|gb|ET185946.1
TTAGAGCCTTCTTATTTGAGATTTACTCTAGAGATGAGATATCTTGAGAAATTTTCTCATTTATTAGTTTAAGCTTGT
CGTGTCAATGTTGCAGTCCAATACAAGCCTATGCAAAATTGGAATTGCTTTATCAATTATGCCTTTATCTAATGATC
CTTCCATCATCATTTTCTGAGCTACCATCAACATGAGTTCAGTGGAACTCATTTCTACAATACTTTTCTTCTTAGTAG
ACTTCAAAATTGTATTGTCAATTGGAAAA
>gi|162316885|gb|ET185579.1
TGAATGCAATATTGAAAGTAAAATGAACCCGACCCTTAGATGTAGTATCTTTTTAGGTGTAGGTTGTTCATCGAGTT
AAAGAGTGGCTCTCCTTCTATTGTGGACAAACCATAGGCATTGTTGTTGATAATTTTTGTGATAATGAATGGTCCTT
TCCAATTTGGTTCAATTTTTCTTGATTTGTCTCACTCTAATAAGTTTCTCTGGTTCTCTTTGAAGACAAGCTCTCCTAT
TTCAAAGTGACAAGGTTTTACTCTTTTATTGTAGCTTCTACTCATTTAGTTTTGATAACCTTGTAAGTGATCTAGAGT
AATCTACCTCTTTTCATCCAAGAGTTCTAACTCTTGTAGCCTTGCAACTCTATGATCTTCATCAGATATGACATGTTTT
AATGAGATCCTTAATGATGGCAATTCTACTTCAATGGGGCAAAATTTCTTTTGTACCATATACCAAGGAGTATGGT
GTAGCATCCATTGTTGTGTGTATACTTGTTCTGTAAGCCCATAATGTAGGATTGAGTTGTATGTGCCAATCTTGTCT
TGTTTCATTAACAACATTTTTCAAAATGTTAAGGATTGTTTTGTTTATTGCTTCTACCTAACCATTGCTTTGAGGATAC
TATCGAGTTGCAAAATGTTTTTGAATGTGGAATTGTATGCAAAGCTTAGTTACTTCATGGTTTTTAAAAGGTCTACC
ATTATCTGTCACAATGCTCATTGGTATGCCATATTGATAGATGATGTAGTTTAGAATGAACTTTTCTATTTGTTTGCC

CATAATATAAGTCATAGAGAATACCTTGACCCATTTAGTGAAATATTTTGTGGCAGTGAGAATGAACTTGTGGCCA
TTGGATAATGTAAGGTTTATT
>gi|162316855|gb|ET185549.1
TCGAGTGCACCTTGGTGTGCACCCCGGTGCATGACCTCGAGTGCACCTTGGTACGTTGCCCCTTGCGTACACAGCG
CCCCGAGTGCGTGCTCGGGTGCACGCTCGCGGTGTCTGTGCACCTTGGTGCACGCACCGCAGCCCCGAGTGCATC
TTGGTGTGCACCCCCGTGCCTGCACAACTACTCGGGCACGCACTGCGCTCCCATTGCGTGCTCGGGGTGCCTCGAG
TTCATTGGTGCGTGCGTCGTTCCTCGATGCGGAGCATGCGTTCGGGGTGGGGGTGTGC
>gi|162316848|gb|ET185542.1
TAAGCCTTAGGTATGCAATCTCCGTGTGGTGTTACAAAGAGAATTCCAGCACCAGAACCACCATGTGTGAAAGAG
CCATCAAAATATAAAGTCCAGAGATTTGAAGATTCCATGTTGAATATTGACTCATCAGGTAACTCTGTGAGAAGTG
GATGATCATCTGTGACAGGAGCATCAGCAAGTTGATCCGCTATAGATTGTCCTTTGATTGCTTTTCTTTCTACATATT
CAATGTCAAACTCACTGAGAATCATGACCCATTTAGCCAGTCGTCCTGTAAGGGTTGCTTTACTCAAAAGATATTTA
AGTGGATCAATTTTTGCAATGAGCTTTGTTTGGTGTGTAAGCATGTAATGTCGCAATTTTTGTGAGGCAAAAACTA
>gi|162316834|gb|ET185528.1
TAAGGAAGAAAGTCTTAGAGGTCAATTTGATGATATGAGGATGGTTGATGGTGAAAATGTTCAACAATATGGTCA
AAGACTTAAGGAAATAATTAGAGAAATCAAAAGTGCTAGAGAAAAAGTGGATGATTCAACAGTGGTTAGCAAAA
TTCTAAGAACTCTCCTACCAAAGTATGGCATAAGAGTAGCAACCATTCAAGAACTTAGAGCCATTGACAAAAATAA
GGTAACTCTTGATTCCATTATTGGACATGATGGTACAACTCAAAAGGTAGAATTTGCATTTAAAGCCTCTGCATCTT
CTCCATCGATAAGGAAAAATAGAGACACTAATCATGAATCTACACACAATAAACAAAAAGATGATGAGGAGAGCA
TGTTGGAACTTGAAGCATTGTTGGCTAAGCGGTTACCTAGAGGTTCCGGTAAGTATAAAGGCAAACTACCATTGA
AATGTTTTGCATGTAATCGGACAGGACATATTGATGCTAACTATCCTAATGAAGACAA
>gi|162316812|gb|ET185506.1
TCATCCACGCGACTTGAAAGCTTCATCTTTCATCTTCCAACCCTCATCCATGTGGCTTGAAATTTCGCCTTAGGTAAA
TCTGCCCTGTCATTTTTAGAGAATTCGCCTTAGTTGTTTGACTCATCTCTTTGGAGGTTCGAGTTTTGCCATTACTGA
TTTCATCCTTCAGCTATGTATGTTGGAGTCCGTTTCATCTCAGATGTTTGGAAGTTCGAATTTCAACTAGTCTTTTCC
AGTGAACTTGGTATCATGCACAACTACATCTTGCTGAATAATTCGCCAACTTTCAGCCCTGCAGTTTGAGGATTTCA
TCTTTCTCCCTTCTTTCTAATGAGCTTCATCTTTCGCTATGTTCAATCTCAATGTGCAGAAGTTCACCAACCTATATCC
TTAGGAGTTGATATGTAGCTTTGATATTTAGATGGCTTTGCCAACTTCAACTTGAGTCAATCTGCAGGTTGTCCTTA
AAAGACAATCATTTGAAGTGAAGTTTGTTATTTTAAAACTTTTGGATCATATCAATTTTACTTGTCAAGACCCCTTTG
AGGCTGAACTGTTTCTGGATCACAACCCTATACAACTAAGCAAAACAAGAAAAGGAGGGTCCCCATTTGCAATGG
GGTGATGTGTGAAGAAGTAACAACAAGACTTTATTTAGGTACTATAGATGGCATTTGCACCATGCTTGTGTTATTT
ATGGCATTAAACCTCATTTAAATTATTTAGGTATAGGTTATAAGCATTGTGATCTTTTTAAATTTGAACAATAAGAT
GAGGTTTAAATATTTCTAAAATGGGTTTGAGTGAAAAAATTATGGAAAACAGAAGAGACAACATACATAA
>gi|162316798|gb|ET185492.1
TTTTCAACATTTGGGAAGGACCTAGGGTCTCCTTCTTATTGCAAGAAGAGCAATTTGACAGTTAGGGAGATGCATA
AATTGGTTAGTTTAAGGATATGACTACCTTCCTAAGGGATGGTTATTATACTCATCATTTTGAAAGAACCAAGAGA
AGACATCTCATGTTGCAATCCATCCCCTATGCCTTGGTGGATGACATTTTATTCAAAAGAGACCTAAATTGTGTCTT
GTTGAGATTCATAAAAGGATATCAAACCAACAAGTTGTTAGAGCAATTCCATAATGGGCCCTCAAGTGACCATTTC
TCGGCAAGAACTACAACTATGAAGATCATAAGAGCATGCTATTATTTTCCTTATCTATTTAATGATGTCCATAGGTG
GATTAGACAATGCAAAGAGTGTGCTCTCTTTGTAGGAAAGCAAAGGTTAGCTTCTCTTCCTCTTCATCCCATTTCAA
CTAATCAACCCTTTGCACAATGCACCCTTGACTTCATTGGTCCTATTAATCTATTGTCAAGTTTAGGCCATAAGTGGA
TTTTGGCCACCATAGACTACTTTAACAAGTAAATAGAGGCAATTCCCTTGAAAGATTTAACAAATAACTTAGTGGTT
CAATTTCTTGAGGGGATTGCAACTACATTTGTAGCCCCTTCCACAATCATATTAGATAATGTGAAGGAATTTTTAGG
ATCTCACATCAACTTATGGACAGTCCA
>gi|162316781|gb|ET185475.1
TATGGTCGCTGAAACGTATCCGACCTTTGACCAACGATTCCAAACGAATTTTCCGTCGACGTGGCGTTTTGTTGGC
GTATTTTGTACGAATCATGTCGCGCACGATTTTCGAAAAACGTATTTTATCATTTCATTTCGTTTCGCCCTAAAACGA
AAAGGGAAAATCAACAAATGTAACGCATGTGAAGGCAGTAAAAACCCACGTCGCGCAGCAAGGAAAAAACCCGC

GACGTGAACGTGGAGAAAGACAAACTTCGCGGCAGGAGCAGAAGTAGGAGGAAGACGAACTTCGCGGCAGGAG
CAAGACGAACTTCGCGGCAGGAGGGAGCAGAAGCAGGTCGCGACGGGAGGAAGGAGAAGGAGCACTTCGCGG
CAGGAGCAGAAGCACTTCGCGGCTGGAGGAAGGAGCAGCAGGTCGCGGGCAGAAGGAGAAGCAGGTCGCGAC
GGGAGGAGAGAAGCAGGTCGCGGGCAGAAGGAGAGAAGCAGGTCGCGGGCAGAAGGAGAAGGTCGCAACGG
CAACAGGTATGTAAATTTTATCCTGTTTCAAATTTTTTAATTTATTATAGTTTATAAAGTTTTATAGTTTATAAATTAT
AGTATGTAAATTTTAATTTGTTTCAAATTTTTTAATTTTTTATAGTTTATAAATTATAAATTTATAGTTTATGATTTACA
TTTAAATGTTAGATTTAAATTTTAAATATATAACATTTATAATTTTATTGATATTTATGATTAATAATTAAATTTATAA
TTTTATAATAATGTTAGATATTTAAATTTAATTTAATGTTCATAATATTTAAATAAAAATTAAAAAATATATATGGAT
AAATAATTTTTTACAAATTTTAAAATTACAAAATAATTAAAAATAATCATAAATACAAATTTAAATTTAAATA
>gi|162316713|gb|ET185407.1
TCTTTATTTTTTAATGAATTTTTTTAATATTATTTTTAAAATTTTTTAATAAATTTTAATTATACTTTTAGTAAATTTTTA
TTGTAATAAAAGTTGTTGATTTAATATTCATATTTATTGATTTAATGTTCTTTTTTTACAACATTGCACCAGTAGTTGT
GAATTTAATACCCATAGTTGAATGAAATGTATACCTACTTCCAAAAACCAAACAATCATGTGATGCCACATGTGCTA
CACAAGTATGGAGGCCCCTTGTGCACGACTATTGGTCTCACCTTTTTTTGAGGCTTTTTTGGACACCTTCGCAAAAA
GCATGCTGGTGTGGTGTCATATTTGATGGTGTGGCCCTGAAACCTTAGTTATAAGCAGGGGACTTGCCAACTAAGT
TGCTATAAAAAATTAAGGGTAGTTTGAAATTTCTATGTAGGATGGAAGCACAAAATTAGGGTGATCAACGACAAG
GTCCTCTCCCCTATTTATCACATCTAAGTTTGTACCTCCCATTAGCTTGGATTGGCATAAGCCTCTAATGAACTTTGT
AGTTTGCACCTCCTTAA
>gi|162316698|gb|ET185938.1
CATTCTCTATATATGGCATAGTAAATATTGTATATATATACCTGCACACTGTAATTAATGATGCATTGCTGTTTAGAC
TGCTTAACATAGCGTAGTGTTGGTAAACAGATTTGTCACTGCGATTGTTTCTCTGAAATTGACCTTGTTTGCTCAAA
GAGCAGTGAGAACTGCACCAACAGTTGGAGAGATTGCAATCAAACGGCAATCTTCCTCTGCATTTGAGAGGGGCA
GCTCCCTCGTATGATTGGGGTATTTATGGTTTTTGTGTACTTTTAACCTGAAAGTACTAAAAGGAGGAGAGACAAA
ATAAATGGATGATAGAAACCCTGATAGTAGACATATGAATACCAAACAGGACTTCACGAATGATAATAGATTATA
GGGGGTTTAAGCACAAAATAAGCTTTGATATGCAATGACAAATAAAGAATGATAACAAATCTGAACATACGAGGG
GATGAGTTACCTTGACACATCCAAACCAAGATACCAAGAAATGATTGTGTTTCATCACATCAATTACATAAACTACC
AATGATTTGCATATATACATAAAGGGATTACTAATTAAATATATGAGAAATTAGCAATAATGACTGAGAAAGACAT
TTAGAGCATACGAGCAAATGCATCCTTTATTATCTTTGTGAGATTATACAATCTCATAAACCCTTCTATACATAGTGC
AAAACCAAAATTCATTACATTTTATCA
>gi|162316689|gb|ET185929.1
TGCCTTCCTCCCGGAACTACCTCCCTAACTATCTTGCCCCGGCCCCCTTCTTAATTAGTTCAAACCGAGAACTAACAC
TAGAAACTGTGAGAGAAGAGGTCGCCCTTTAGAATAGTAGGGGCGGATGGATGGGCCCTGCTAAAAAGAGAATC
TAGTCTCCCTGCCTCCCAACAACTATAGGAAGGGAATTCTCTGAACCTGCAGGGGCCCTTATAGATAGTAGGGCG
GAATGGGCTCAGGCCGTCCTCTGCCTACTATTATAAGGGGCTCCGCCCCACATGAACTCCCATCCATCCGAAAGCA
GATAACTACCGAAAGCAGATAACTATAGTTCTTTCCGATGAAGAGA
>gi|162316668|gb|ET185908.1
TAGGGTAAATTAACTTTTCTTGAAGTGTCTTGTGCTTTTGGCACACTTTTTGTTTAGATTAGAACCCTTTGGTGATAA
CAAAAGTATCCTCTCCCCTTTCCTTCCATTGGACTTTGGTTGTAAGAGAAATTTTTGTTATCCTTTTAGAGGGACTAC
CTCATGAGAAGCCCTTAGGGCCATGTGAGAGGAAATGACCTAAGTGGTACTTTGTTTTAGTCCACTATATAAATAA
TTATGCTTTTAGCTGAAAGTATCTTATCACCTGGTGTTTATGTGTTATATCGATGGATGTCTCCCTTAGTAATTTTTT
GATGTGTAATCCACATAGTGATGCGTAAAAACCCATAAGGGTTGGGAGGCCTCATAATTGAATGAATCATTGCAT
GTATAGCCACTAGTATACCCCGAAACATGACTAGACACCTTAGATTAGTGGCCCACCCTTTTTTGTCTAAACATTAC
AATAAA
>gi|162316660|gb|ET185900.1
TGTGAAACCACGTGGTTCCACATAGGGTCATGACCCCTGTGTGGAGCCACAAACCTTCTACACCAGCTATTACCTT
AATCAAACTAATGAAGTTGCATAACAAATTTGTTAGTTTAACTCTTAACTAAATTCCAATCTTAATTAAATCGCAAAT
AACAAAATTATTATTTTGTGTGCTGAGACGATTAGAGAACAGAATTAGTTTTCTGTGTGTTACAACTGAGACTAA
>gi|162316659|gb|ET185899.1

TTAGATGTAGTAGATGTTGCTGACCTATTTTTCAAGGAAGTAGTGAGATTACATGGATTACCTAAGAGCATCGTTTT
AGACAGAGACACTAAGTTTGTTGGATATTTTTTTAGAACACTTTGGAAGAAGATGAAGATAGCTTTGAAGTTTAGT
TCTACTTTTCACCCATAGACCAATGGACAGATAGAAGTAATGAATAAGAGTTTGGGAAACTTGTTAAGATGCTTAG
TTAGAGACAAAACTAGAAGTTGGGACTTGATTCTTGCTCGAGTAGAATTTGCCTACAATAATTCAATGAATAGGAG
TATCCGAAGAACACCTTTTGAGATTGTTACTGAAGCACACCCTAGAGGCATATCAGAATTGAGAGACATCAGTAAT
GAAGATAGGAGTGCAAAAGCAGAAGAATTTGCAGAGCATATGAAGACATTCCATACTCAGGTCAAACAACATTTA
GAAGATATGAATAGCTAGTATAAGGGGAAAGCAGATGAAAAGAGGAGACATAAGGAATTTGAAGTTCGGGACA
AAGTGATGGTGTATCTGAGAATTTTTTTTTTCCAACTGGAACCTATAACAAGTTGAAGATGAGGAAGTTTGGACCT
TGTAAGATCTTGAAGAAATTCAGTTCCACAAATGCATATGAAGTGGA
>gi|162316634|gb|ET185874.1
TCACAAAAAGAGGATGAACCTCTTGAAGATTGTATTGGGAGATTTTAGTTTTGCTTCAATAAATTACATCACAAGCT
CTCCTCGGAATCTCTCAAGTTGGTATTTTTTAGAAGACTAAATAAAGACTACATAGATTCCCTAAACCTTATTGGAG
TATGAGACATCTCCCAATTATTCTTCAAAGAAATCTATCATATTATCATAAACTACTTCTAATCAACTCTAAGAAAAT
GAAAGAGCTCTAGACTCATGCCTACCATTAAGACATATCCTAGTGACTCTAGAGTGGAGCTTAGAAATATTCTCTC
CAATGTGAAGGAAGAAATTATTAATCAA
>gi|162316590|gb|ET185830.1
TTATCCTTTCATACTTCCAGGTTAGAAGCACAGAAACACTAAAATAACACTATAGGAGGGGCATCAGCCCTCTCAA
ATATAGAGGGTGATCACTTATTATGTGTGATTTCTCCTATGGCATATTTTTTTGTTATAATCACTTGTTCTGTGTGAT
TTCTCCTATAGCATTTTTTTATGCACGAGTCAAATGAAATTCAGCAAAGGTTTAAGCCAAAAAA
>gi|162316579|gb|ET185819.1
TTGGGATCCAATTGGTTTGGGCCATCTATACGAATCTTAATAAGCTATGGGTACAAATTTTAATGGAAAAATATTTG
GATATTCATGAGGATTGGAGGATCCTCACAATTTTAAACCCACCACATGGATCAACCATATGTAATTTGATTGTGTA
TTGTAGGAATGTTGTTATAGAACATGTAACTTGGCACATTGGGGATGGAAGAGATGTTAATTTTTGGTTGGAATCA
TGGGATGGAAAGGAAATTCTAGGTAATTTTGGTGATGTGATCAAGCTTAGAGGTATTATATGTGATATTTAGGGG
ACAAAGGTTAGAGACTAAATGGAGCCCAAATTTATAGATGGTAGATTGGAATGGGATTGGAAGTCAATAGATGG
GGTCTTGCATGATCCAA
>gi|162316566|gb|ET185806.1
TTAATAAAAAGGGAAAAACCAAGCACACAATGGAAGCTAGAACACTTCACCTTTTGTCTGTTCTTAGGATACTGCA
TTTGGAGTTTCTATCTGATACAATTGCTCACGTTCTTGTACACATATGCCCCCTTGGAGTCATGTAATATTCATTTTG
GAGTTGTAAGACATATGTGCAACTAAATGAGGTAATATAGCATGTTGTTTACGCAAGTTTTCTTATCAAGACACAC
TAACAGAAGACTGCAGAAATGTTACATAAGTCCCACAGTGTTCTTGGAGGCTCTATTGGAGCAGCTATCTTACAGA
TGTTGCATGATGGCAGAGGAAATAAGAAGGTAAAATCCCTGCTCTTCTAGATTTTTCTCACATTGGGTTTTTCCAGG
GTAAACTCTTGTATTCTCTTGTATTGTATTGGTTTGTTGTATCAGTCCTTAACTTACTTCAAA
>gi|162316562|gb|ET185802.1
TTAGTTGATCGATTAAAAAGTTATTTAAGTATCTATCTTCCTTAATTGAATATATTTTAGGACTATAGGCTTTGGGGA
GAGACCAAAGTACCTTTTTCACCACAAACAATTCTTTCAATGCTTTAGTAAGACCTTTGATTGCATTGACAACTTCAT
CTAGCCAGAGTGGATAGCTAGCAATATCCTCATCTTCTAACATCCTTTGGTTTTCAAATTTACCTATGTATATCTATA
GCTTGGATTAATTGGATTTAATATCCCCTTCATAAATACTAGTAAGCTTCTCCTAGATATCCTTATCAATGCAATTGT
GCATCACCTGTGTA
>gi|162316550|gb|ET185790.1
TATTTGCTTATTTCAGTATTCTTCAGCTCAGTGTCCCAGTTTATTTGTGGTATGCCTTCCTAAAATCATTTAGGGCTTT
TTGATACACACTTACTATTTTTAGTAAGTCACTTGGATACATTTGCTCCATACTATTTATAGTAAGTCAGAGAATGGT
CAGTTGGCATCATGTCTATTTTGGGAATGGAGGTGAATTATCACCATGGTTGGTTTTGGCATTCATCATGGTTTGG
AGAATTAATTAAGATATCGTTATTATTAAGTTAAATTAATGTTTTAAATTTAATGATATTAATGAAATAAAGTGACTT
TATGATACGATGGCATAAAGGGGTAAAAGTGACTTAAATAATATACGTATCAGAAAGTAACTTATTTAAAGTGATT
ATGACCACCAAATTTATGAAGTTGAAAAGACATCCACAGTAAATAATTAAAATGATTTAAAAAGTCACCTTGGGTG
TGGGTTTTGAGGAGGCATAAAAGCATACTTAATGCTTTCTCATTTTCATTCTTGATCGCATATGAAAGAAGAGCTG

AGAAAAGTTTACTAGTTGTGTTGAGAGAAATGGCTTTTTGAGGACGAAATCCCTCAAGAGGAATGGTATCTGCAT
GTTACGAGACTTTTTCCCGTGGAAAA
>gi|162316525|gb|ET185765.1
TAATTCAAACTGGGTTGTTAATTGGGCCATGGGCTAATCCATCTCCAAATTAGTTAGAGGAAGTTGAATGGTAGAAA
CCTGATACGGGGTGGATTAAGATTAATTTTGATGGGGCTTTGAAAGGTAACCCCAAACCCTTGAGAGCAGGATGT
GTGGCAATGGATGATAAGGGGAATAATTTGGCAATTTGTGCTCAAAAACTGCTACAAGGAACAAACAATGAAGTA
GAGGCCAGTGCAACACTACTAGTAATCAAAATGGCCCATAAAATGTCAATCCCACAAGTTCACCTGGAAGGAGAC
TCACAA
>gi|162316524|gb|ET185764.1
TATTTGACCTACCCTTGGAGATGTTGCACTCAATGACATAGGCAACAAATAAAATGATTTGCCTCCCCATTAGACAT
CAATGTTGTCAATGTCACAAATGGTCTTCTTCGTATTGATATATAGTGGTTCATACTCCCATATTCTTGATAGATGCC
CTCGAAGGGGACCCAAGACCATAGGAGTTAGATGGAGAGGAGGGTTAGTGAGAAGGCCGACATGAGAAGATGA
TATAGAGGTAAGAGGAGGATATCCTCCTCAACAATAGTCTTGATCATCTTGGTCATTATTCCAATAGGTAACCACA
CCAAGGTTCCATTTGTGGTAATTCCATCATAGGTACCATAGACCAAGGTGTCCATAGTTGTAATGATAGGGACATC
AAGTGCATTAGAAAGAAAGGTGGCATTTGTGGATCTAAATTGGTGACCTAGTTACCCAAGTATGAGTTTAACTTTG
ATATACATTCTAAACTTCCATGTGTCTTCACTATTACCATAATGTGGTATAAATACATGCCCTCTCCTTTATATTTGTA
ATTACATATGAGAGTGGATCATCTTCCTCAATTGAGAACTAACAAAGATGGTACTAACTATGCAAACACAAGGTCA
TGTCTTAGATGCCTATAAGCCCCAACAAATGACTAATAAGAACATCTAGCTGTTTGATCCTATAGCATGAATCAAAA
TGA
>gi|162316516|gb|ET185756.1
CTCTATTTTCTTTGTGAAATGGAGAGATTGAATGCCTAAAATTGATAATTTCACCTTGAATAAGGTTATTATGCCCA
AATATTTTTTCCAAATTTAGTATATTATGTATTCTAAAAAAATACCCAGTTTGGGTATTGTTTCCAAAATTTTATATTT
TTAAAATGGGGAATTTAAATTAGTTTAAAATCTTAAATTATTGATAAAATTATGTGGGGTTCATATTTCATGTTTTGT
TAAAACGTATTTAAGAAAAAAAAAATGTTTTTTGTAAACAAAAAAAAATCATTTGGAAGAAAAAACAACAAAAAAA
AAAAATTCAAAACAAAAAATGAAATGAAAACGTTTTTTTATAAAATCAAATCAAACATAATGGAATCGTGTTTTTAT
TTTTTGGCAGGGTGGAAGGGGAGCCGAGGGCTCTACCTGTGCCACGCAGTGGGGAAACCCTAGTGGGAGTGTCC
ACAATGTGGTGGCGATGCCCCCACATTGTGGGGAGCGTCACCCACTATGGGGAGTGCTACCACAATATGGTGTAT
TGTGCCCCATGCCCAAACCCTAAGCTATGTGTTGAAAATAAAATATGCCCAAAACAAAAACAAAAAAAAAAAAGCG
CAACTCTCATTTGCTTTGGTAAATACTTAAAATCA
>gi|162316514|gb|ET185754.1
TTTTTTTATTTTAAAGTTCAGAATCAATTATATTGAAGATCTTAAAGACAATGTTGTTGGCTGAAGGCTTCATAAAG
TAATTAGTAAAAGGTAAGAAATTCCCAATAGGTGCGATTTATGAGAAGTGGCCATAAACAGAAATTACCATTGTCT
TATTAATAATAAATACAGCAGCGATCAAGGAAAGAATGATTACCTTCATGAAGTGGCACGATATTATGAAGAGGC
CATGTCAACAATGAATTAGTAATGGAATGGTATGATTAAGTGGTGAA
>gi|162316512|gb|ET185752.1
TTATTTATGTATTTGGACAGGGTTGCAGCTTGATGACATGTTCACTTCTACAGGTGTGGGTATTTGAGCACATTACA
TGCACTAGGCCTATCGGCTATCCCATAGAGATAGCTAGCAAGAGGCCTAGGGTGTTCGCCTATCCACTTACCAACG
AGTGGAGATTCAGAGATTTGCTACATTGGAGAGTGACACTCGACAGATTGACAGTAGAGATGATAGTGTGGAGA
CCATATCTGCAGATGTATAGATGGGAGGGGATGGTTAGATAGTTAGCATGCTTACAGAGGAACCACCTACTGAGA
AGATGACATCCACACATCATAGTCCCATTCTACTTTGATTGAGTATGGAGGCAGTTTGGAATAAAGCAGGGTGTTC
CAGCCAATGTGCCCATCTATACTCGACACTCATGAGTCCTTCCTAGACCCAGAGCAATAGCAAGGCCTACGATAGA
TGATGTCAAGATTTTAGATATAGAGGGGTCTGATCAGGATGTAGCCACCGATTACTTGAA
>gi|162316506|gb|ET185746.1
TTATTGTTTAAGAGAGGAAGCTAAATGTTGAAGTTAATAATAGAATAATAAATATTATTACTCTTACCTCAGAAGAT
TCTTATTATTTTATTACTTTTTTTAACAACAGATTGCTCAAAAAATTGTTGTTCTTGTCTATTATTAGTTGGCACTTGG
CTGGATATACCTTCAAAAGCTTTTGGCCATTCCTCTCTATGTTTAAGATAGGAAGCTAAATGTTGAAGATAAGTTAT
ACAACTTTCTGGTATATTTAGACTAAAACATGTATTTGAGATCTTCCCATGATAGGAAATTTCCTGGTGCTGCAACA
AAACCTTAGAAACAATTCTTCTTTGAATCAATCCCAATTGCAGCACCAATAATCTTCTCTGAAAAAGGTCAATCTCA

ACCATTCTGTATACAATTAACAAAAGCTCCAGAACAGTCCATCAAAGATTATGCATTTGACAGCCATTATCTACCAA
ACCAAACTAGCTTTATGGGCGAAACACTCGGAACCATCCCAAATTTTAGATGGTTCCTACTTATATATGGAAGGCTT
AAACAGTAGACAATCTAACTCATTTGGATGGTTTTTTGGGGTTCTTTTAAATTTTATTTTGATGAACAAAATGCCAA
AGCACCAGGGCATCAGGTCATTCCCTTAGTTCGCGTGATTGTCATAGAAAACAGGATTAAATTGTTGTGAGTAGTG
ACTTCACCAGATTATCAAAGTTTTGCGTATTTGCAAGTGGAATCAGATCAACTTGATCTAAATTTGATTGGTACTCA
TGACTCAATTGCTGTNCAGGGTAGCCCTGAATTTAGAGATTTAGCTGCGGTAACAGTACTTTGATATCCACTTATTA
TTCTAAGATTGATCTGAAATGAAATAGATTCAAAATTTAAGCATAGTTTAGAGTTCATTCTATAGTGCACAACAAGC
ATTGTACAGA
>gi|162316483|gb|ET185723.1
TCATTATCTTAGAGAAGTTCAAGAGAAGAAAGTGGTATTGGAGTATGTTAGAACAAAATTACAAATTGCAGACATC
TTCACCAAACCCTTGCCCAGGGACACTTTTGAGTATCTCAAAAGTCAATTAGGGGTCCTACCCCCATCTTCTACTCA
CTAACAGAGTTCGGTGAAAGCATCAATTCGATGACTCAACAGAATATTTTTATGAGATGATGCCGATTTACACACT
TTAGGAGGTTTTCTAAAGGTGTACATAAGATAGAATAGGGAAACTTAAGTGAAGACCAAATGCTCTAACCGAGAC
TGAGTTGATACATAACAAGCAAGGGAACCACCTCTCACAAGAAGAAATCATGTTTTAATTCTTTTTCTTTTTGGCAT
TGTTGTCAAAGGGGGAGAAGAATAATGACATTGGGAAAAGACTAATGATAGGGGGAGAGCATTTGCCTTTGCAT
TTACATTTCAAAAATTTGGATTTTCACAAACTCAATAGCAATCTAAATCAGTAAGGACAAATCAATTTGTTTTGTCAT
CAATGCCAAAGGGGGAGATTATTGGCATTACAATTACAATGGGAGATTGTTGATATTTCAATAAGGATATTGAGA
AGGTTGTTGATGATTATAGATATAATTGCTTAAGGATGTTTATTATCTTAATGATAA
>gi|162316475|gb|ET185715.1
TAGAAAGAGCTAAGATACAAACAAACTTTGATGCTGCTATATCTCTCAATGAATTTCTAAAGAAGACAAATGAGGT
TATTTCCAAAGAAAATAAAGATACCAAGGAGATTCTTGAAAAATTCAATAAGAGCACAATCAAGCTTGATGAAAAA
CTAAAATCTATGACACCAAAATGTGACACCAGTGGATTGGGTTATTCATCGTATGAAAAATGTGAATCTTCTAAAA
CAAAAGATGTCCCATCTAATGACAGACTGGTATCTATAAGTAGAAAACTAGGTAAGAACTATAAACCAATTTTTTTC
AATTGTCATAAAGTGGGACACACTGCAAATCCCTATAGAAGCAAGCCTAAAACTGTCAATACTAATAGATCTAAGG
AACATAATGGATATATCAATAGATCTATAAATTTCAATGGTTATTGTTATGCATCCAACAAGTTTGGACATAGAGAT
TTTGAGTGCAGATCTTCTATGAACTTTCGAAGGCCTAACACGTCAAGAACTAAAGGAGTTATGCATAATCCATAAT
GGAGCCAAAATTTTGGATGGACCAATACTAACCAATCAAATGGTTATAAGATATCAAAAGGATGTAATGTAACAT
GCCTAATCTACA
>gi|162316471|gb|ET185711.1
TAAAGGATTATAAGCAACCTCACTCAGACGTACATCAAGAATTAGGTGAGAGTTTTGATGATGCTGATCAAGAGG
AATCTATTTCAAATGAATCATCTCTTGTTGCCACTTCTTCTTTTAATAAGCCAAATCCTTTTTATATATCCTCGTATATT
GTCAGTTGTAAGTTCAGCAATTGCATTATTGATTCTGGTGCATCTAATAATGTAATGCCTTCTAAAGTGGCTCATGC
TCTAGGCTTATCTTCGAACAAGCCTACTGGTAAAGTGTTTTCAATGGAATCAAAACAAGTACCTCTCATTGGACAAA
TAAAAGATGCTCAAGTTGCCTTGGTTGCCCATCCTCAAAAGAAGTTGAAACTCAATATTTTAGTTGTTGATATTCCA
GCCAGTTATGGTATGTTATTAAGTCAGAGTTTTTGTAAAGATCTTGGTGGTGAAATCCAATTGGATTGGTCTCATGC
TTTGATACCACTTGGCAACAAAAAGGTTTGATTAGACCCAAATCCA
>gi|162316464|gb|ET185704.1
TATTGAGCATCTTGATCTAGAATCCACATATTTGAGCTAATGAAGTCACCCCTAGATAGTGGTGCCTACTATCATTG
ACTGACACTTTCAAACCCAGATTGTAGACATAGGCCCCTCTTTGTATTCTATTTTCAGATCTAAATTTATTTTTTGTTT
TGCAATTTTGTATCTTTCTATTCATGTAGTTTCTTGTCAATTTCCTTACTCTTTACATATTTCTTGCATTTAATCAATCA
ATCAATCAGAACCCCTAAATGGAGTATAATAACATACACTATCCATCTCTTTGTTAAGGATTGAGGTTGGATCTTAT
TGATTATCCTTCAATGAAAAACAAGTGGGAATAGTGATCACCTAAGTTCTTACTAGTTTATATTTCTAAGCTAAGAA
TCCTATTCCCACTACATTTCAATTATTTAGAAATCAACAAAAAACTACAATATACTTTCAAATAAATATTCTCACATG
AGTATCATAATACAAAGATCCATAGGTAACTTAACAACTATAATATTCTTACTCGCGACCTTCATCTCACACACCTAA
TA
>gi|162316432|gb|ET185672.1
TGTTGTAAAACTCATGCACTAGAGAGAGAAATTTTGAATTTTGAATAATGCAAGCAATTAATGAGATAATGATTAT
CCAATTTAAATTTGCACAAGGAGAGGGAATTCACAACTTACAAGAAGCACAATTGGAAACTTAAGGCCAAGAGGA

TAGCTCTTAGTTTTAATTATTGCTTTGAAAATTGAAATCTTGAATTTTAACTTGGTTTTCAAAATTAGAGGTAGCAAA
TTTCACTAAAATCAGCCAATATCCCAGATTTAGGCTATTAAATACAATCATAACAATCTCCCGAAATTTCAGGAAAA
ATGTCGAGGACCGTGTGCACGCGTGCACATGGTCCTCACAACTTTTTTGAAATTTTAGGGGGTGAAAGTTATAAT
GATTTTAGAGCTAAA
>gi|162316354|gb|ET185594.1
TAGTTTGCTTAATTAGAAACCTATAAGAGGTTGGGAAGACCAACCAATGGGAACTCACATCTTGTCCAAGAAGGTT
GGAAAGAGTGAGGGGAGTCTACTTAGAAAGAATTTTGAAAATTTGTAGGTTGTTCACCCACTCTTCATCTTTGAAG
ACTTAGTAATTTTGAAAATGGTTTTTGAGAGTAGGGGAGAAACTTCAGTGTTAGTAGTAGATTGTAGATTGGGAA
ACTCTCCCTCAACTCATCTACCTTCAGACCTAGAGTTGAGGTTGGTAATTCCTTTCTCTTAGCATGTTTTCAATTTCCA
TTGGTTTTGTATAGTGTAGATTGCTTGTTGAAAGAAATGTCATAATTGTATTTTGTTTCTAAAAATTTAGTTGGAAT
TTTATTTTGTGTATTTCCATTTATGTATATGGGTGTACAAATCCAATCTTACCTTTGGTAAGGAAAGATTGAGCCTTG
AGGTGAAGGGATTTTGTGACAGCCCATTGAGTGTAGAGTCTCTCTTCTCAAAGAAGATAGGGATAAGCAAGGGGT
ATGGGACCCTTGTATCTCTCATTGCTTTGAGCAATGTTGGGAGTAATAAGGGCATTGTATGGTTAAGTGAGTGACC
ATGGGATTTTAATTTTATA
>gi|162316353|gb|ET185593.1
TTATTCATTAAATAATTAGCTGAAAAATCTAGCATAAACTATATGGTTTTAGCTAATTTCTCATTAGATCAATTTTTA
AAATTCCAAAAAAAATTTGACATTTTAGGGGGAGCACACCACGTGCTATGTTATGTTTTTTCTAAAAAAACAGGAC
CACTTTGCGTGCTCCTCCTTTTTAAAACCCTTAACCTCCACCATTTTCAAAAAAACTAGTATTTTAGAAAGTAGACTC
AGAGCACTACAACTCTTGTTCTTGTTGCATCCTCATATTTTGAGTGTAACTATCTTCATTTTCTTGTCGAAATATAGA
CTTTGCTCATTTCAGAGAAAAAAAAAAGTGGCATCTTAAGACCCCTTTTTGGTCCCCCATCTTTGTGCCCTTACCCTTA
TATTTATTTTTATATTCATCCCGAATTGCATTTGATGGAGCTAATCACTATATTTAGTCTTCACATGAATAAATTTTCA
CTTCGTTTAGCTAATTTGTTGCATTTTTCATCACTAAACAAATATGCAAATCAAATTGTGA
>gi|162316351|gb|ET185591.1
TATAATTATTATTAATTATAGTTATAATTATTAATAATATAATAATATATTAATATTATATTAATATATGATCATTAAT
ATTTAATTATTTAATTTTATAATAAAGTATGGTGTCCCTTGGTGAAAGGGCATCTCTTGCATATATATGATGAGATG
TGTGATCTCATTGAGTGTAGTAGTAATAATAAAAAATAAATAAAATAAAGGCAATACTTGGGACTTTAACATGGTA
TCGGAGTGGGTTTATTTAGAGATAGCCTTATAGCTTAAAAGAAGTATACATGATTATTGGAGATTCAATGTAGCTA
GGATGAACATACATAAACAGTGACAAGTATGGTTTGTTATGGGTTTAAGGACTATCAAGTTTTGTTGTCTCTGTCA
AAGAGAGGCCTAAGAATTATTTCAAATCATATACAAACTTTAGACCCTAAGCATCTGTGTATTCATAAGGAAGCTT
AA
>gi|162316350|gb|ET185590.1
TTTTAAGGTCTTGATAGATAGGGTGTTGGATATTTAATGTGGGTAGACTATGGTTTGTTAGCATAGGCAGGTTTTT
ATAGGTGAACTATGTAGGTGTTTTGGGTGCTTGTACAACATGAAGGGTGCCAAGGAGAAATGGTACTAGCACAAA
GCAAGTCGACACCTAAATTGTAAGGTTTGACACCTATGTAACCCAAAATGATGCATGTGTATGGGTAGAATGGCAC
TTGTGCTAAATAAATTAGTGCATATGTAGAGGGAAAAGGTGCTTACACAAGTGATTTTGGCTCTTATCAAGAGGG
AAAGGGCACCCATACGTTAGAAAAGGGTGCCTACAAAAGTAGGTTTAACACCTACACAAAAGGAAATGATGCTAG
ACAAGCAAGTTTGGTGCCTACATAAAGGGAAATGATGCTTACAAAAGTAGATGTTGACACTTGCATAGTGTGAAA
GGGTGCTTGTACAAGTAGGTTTATTATATGTGGAAAGGGAAAGGGTGTTTTCACTAAACAAAACTTGGTGGTCAT
GCATAGA
>gi|162316344|gb|ET185584.1
TTGTGTGGGGGAAAAGTGACACTAAGTAAACATTCCCTAATCTCACTTTGAATCACACACTTGTGGAATACGAAAG
AGCCTAGAGGTAGTGCACAATTGGCTACTTCTTCTTGTGGAAGAGAGAGCCACAAGCTACCTATCAGGGTTTCTAT
TCCTTTGTTGTAATTGAGAAGTAGAATGATGCAAGTTCAACTATTAACCCTAAAGTGTAAGTATGAACTAGTAACA
AGATTGTAGAATGGAGATTAAATGGTGAAAAGCTATTAACATAGGGATAGGGGAAGAATGTAGATGCATACTTG
GGGTCAAAATTTGAGTGAAAATGTTTGGGACAGGAGTGCATGTGCCACTGACCTAATTTTGCACCTAAAACTACTC
CTTTTTGCAACTTGAAAGCTATCGAAAAGTGCTGAAAATTGCTATTGTTAGAGGGACCAGGGTATCCAGCACCCCT
GTCCTAGGGACCAAGGTGCCCAGTGCCCCTATCCTAGTAGGACCAGGGCACCCCGTGCCCCTGTCCTGGTCTTCTA
AGCTGAAACTTGGTGTATGGCCCTATCTTAGTCTGCTTCTAA

>gi|162316335|gb|ET185389.1
TCACATGCCTAAAGAAAAAAGTTAAGAGTATCACACACCTAAAAAACACATCCTCTTTTAGGTGTATTCCATCAATT
GCCTTTTGTTAGAGGTTGGTCTTTACTATTAAATTACATATGGAAGAGAGGCAATTAACTATCCATAACCTTTCCAA
CATTTTCCTATTTGAAGTCAAGATTGGGGAATGACACATGCCTTAGTTTCCTTTAGTTACCCTAGTTGCCTTTAATTT
GTTCTCATGTGTCTCCTTCATCACTTTTGTCATTAGAGCTCATGAGGGGAGTAATAACCTATTGATGGTTCTCAATTC
CTAAGGAACTAAATGATGGGGAAATGAGTACTCATTAGGTTAAACATGGAAATTATGAATAATTTCAAATCATAAC
ATTCATTGAAGAACAATAAATTAAGCTGAA
>gi|162316332|gb|ET185386.1
TAAGGTGCGTGCACCTTGGCCCAAGTTGGGTGCCAATGGTGCTGGGGTGGGTGCCAAGGTGGGCACCCTGGTGG
GGTGCTAGGGTGTTAGGGTGTGTGCAAAGGTTGGTCACGAGGTCGATGCCAAGGTGGGTGCCAGGGTGGGTGC
TGAGGTAGGATGCAGGGTGGGTGCTAAGGTGGGTCACACAGCTTGGGTGCCAAGGTGGGACGTACAGGTGTGT
AGTGCATGCCTTGACCAAGTTGGGCACCCTGGTGCGTACCCCGGCCAAGGTGGGCACCAAGGTGCACACCTCAGC
CAAGGTGGGCACCGTGGTGCACACCCCAGCCAAGTTGGACACCAAGGTGCACACCTCGACCGAGGTAGGTGTCA
GTGGTACTAAGGTGGGTGCACAAGTGTGTGTCGAGGTGGGTGGGTGCTAGGGTGTGTGCAAAGGTTGGTCACAA
>gi|162316316|gb|ET185370.1
CATTGGCATGGAGGCAGAGATGTGGTTGTTGGATTTGAGTAGGTGTTTTGCTATGCATCCATATAGCAGCAACACC
AAGGCAAGATGTACCATTATGCACCTTCATGATTTTGCATCAACATGGTGGTGCACTGAGGAGAAAAAATTACGAT
TGGAGATCACTACAGTTTCTTGGGAGCTATTCCTTGAGTGGTTTCATGCTCGATTCCTTTCAGAGCATTGGAGGCA
GCATAAGGCTAATGAGTTCCATGACTTGAGGTAGCATAAGATGTTAGTGGATGAGTATGAACATAATTTTTTTGAG
CTCAAACATTATGCGGGCATTGTGGATGATGAGTCGATGCTTATTTAGGATTTTGTGAGAGGACTTAGTGATCATA
TTAGTGGTGAGGTTCAGATGCATGAATCTAAGACCTTGGAGGTAGTTGTAGAGAAGGCAAGATTAGTTGAGGAG
AATCTTTCCTTAGCTAAAGGAGGTGCCATAGGAGGAGTTGCAGTAGGATAGACAGCTAGTGCACTAGTTACAGGT
TTAGTTGTGAGAGGTTCTTAGCCGCAGTTTGCAGGTATTGCTAGGAGCCATCCCTCTTCTTTTTGTAACAACCAATG
ATTTAAGAAGAGGCATTCTTAGGGTAAGAACTTTGGGCAAGGAGATAGATTTTAGGTTGATAGGAGTCATAGTTA
TCAGCATGATAGGAGGTCTAATCCTTCACAACCAGCACAGAGTTCACAACCAGCTTCTAGCAGAGGTAGTGTTCAG
CAGTCAAGTACTACACCAGTGAGTAGAGTGTTTGGCAGGAGAGATTGTTTTACAA
>gi|162316315|gb|ET185369.1
TTAAAAATATGATAAAGGCAAGTATATGCCCCGTCATGTTTTTCCCCAAGTGAGATGACAAAAATTGGAATAATAA
CAAGCCAAATGAGCCTAAAAAAAAGGGGTTGGGATGATTTGGCTATATACTTGAGGGATGTGCCCCAAGAAGGGT
CATTAGCACTATTCATCATGAGCATTTTCCCTGAGATTTGTATAGAGCCTCAACATAATGCCATGCAAATGAGAGCA
CCAAGTGAGCAGTATCGAGGAGCTACACACTACGGTGGAGTAGGG
>gi|162316284|gb|ET185338.1
TGGTTTGCATCACGATATCATCATTTAAGCTGGAGTTTTGTCCGCTGACATTCACATCGGGGGTGCACCGGGTGTA
CATCGAGAAGGCACCCGAGGCGTTTTGCACGCACGACGCTGCGCACCAAGGTGTGCCTAGGGGCGTGCATCGAG
GTGCACACGCGAGGCAAACCGAGCGCACCGAGCCAACACGGAGCATGCACCCGAGCACGCTCGAGGTGTTATGC
ACGCACTGGGGTGCGCACCAAGATGCACTCAGGGCCGTGCATCAGGGTGCGCACGGTGGTCGCCCCTGACGCGT
TGTGCATGCACGGGGGTGCGCACCAAGGTGCACTCGGGGCCGTGCATCGGGGTGCACACCCCCACCCCGAACGC
ATGCTCCGCATCGAGGAACGACGCACGCACCAATGAACTCGAGGCACCCCGAGCACGCAATGGGAGCACA
>gi|162316241|gb|ET185295.1
TTATGTCAAAATTATAGTTGTTTGAAGTTACAAAACTAAGGATCGTCCTTCAAATTTGCTGAGAAAACTGATTTTTT
GGAAAATATGAATCACTTGAGCAAAATATGACTCCACCACTGCAAATCCTTTGAAATTCTACCTCAAATTAAAAAAA
AATTGAACTCCAAAAGGACTTTGTATGCCTAAGATATGACCTTCCAAAATTGAAATCTTCAATGGAGAGGGGTGCA
AAATTTGCCAAATTAACAAACCTCCCTCTAATTTGGGAAAATGCCAATTTTAGTTGAACCGCCAAAATAAAAAACCA
CTAGATGAATTTGGAGTGTGGCTAAAATTAGTGAAACTACCAAATAAACCACTAAATTGGGCATGAAGGTTGAAA
AGCAAGTGCATTA
>gi|162316234|gb|ET185288.1
TAAGGTTTAGCAATAATCAATATTGGCACTTCCAGGAGTATTTTGTGAAACCACTCAGTGCAGCCTTTGGACAGCA
TAGAGATTACACTTTGTCTCCTAAAATTAAAAGATTCCTCAGGCCGAAGGATTTTGCTCCAGAATCCCAAATCGATC

ACAATTGGGGTGACTGGTATTGCCACCCTAATCATAAATAAATTAGAGTATTTGGGTATGAAGGAAAGCCACATAT
GCTTCCAATTACAGTGCCAAATAGGGTGGCTAGCCTAGAGATTGTGAGACAATTGTCTGCTATCAGTGCCAAACAA
>gi|162316222|gb|ET185276.1
CATTTTCTAAAATGATGAAACCTACAATGTAGTGTGGGATGCATTGGCATTCCTATTTCTAGTTGGGATGTATTTGG
AGGGTGAGTATTAGTGAACCAAGTAATGGGATGATACATGCAATAAGGTTACATCCACTTCCTATTGTCAACTAAG
CACCATTTGTGGGTTAATCACTACGTACATGTGTAATAATAGCTACATTTGTTATGTATTGTTATATTTATGTCATTT
TTAGTGATCACAACATGTAGTATGGGGTGCTTGTCTTGTCATAGAAAAAAATGAACTATGAAGATGATGGGACCC
ATGGTTTAGTATAAGTTGAATTGAGATTCCTCTTTCTAGTTGGGGTGCACTTAACGATTCATGTGTTAGTGAACTAG
GTAAAGTAGACTTAAATATAAGCACACATTCTAAAGTAGGCACTTGCTTAAGTGATAGTAGAAACAACCCTTATGT
AACTACACTGGTAGATGATGTCACAAGGTGTCATGTGTTACTACACAAGCTAAGGTTATCCAATAAGGATAAGTCT
TCTCGTTGGATATTAGATTGTGAAGGAGACATTCTTTGGCTAATAAATAGAAAATTTCAAGTCATTCTCATTAGATA
TGTTTGACTCTACAAACTTTTTATTATTGTTATAGTGGAATTCAACAAAAGCTAGGGAATAATGGAAGTTGAAGCAT
TAGGTGCCTTGAAGATTTAGTGACATATAGGTGATAGGAAATATAACTTTTCAA
>gi|162316212|gb|ET185266.1
TTGTATCTAGCTTCCAGACTCATCGTTTGACAGTGGGAAGTTCTTATGTTATTCCTTCTTTTGATGCTTTCACAGAGA
TGTTAATATTGGAACAAACTAAGTTGTTGAACATGGGGTTACTCAACTCTTCAAAGTCCAAGGCTTTGGTAGCAAA
TCAAGAAAATCAAAGTAATCAAGGAAGTCAAGGCAAAAATTCCAACAAGAAGAAAAAGAAGCAATCTAAGTCTCA
ACCACAACAGGAAAAGGGCCAATCATCCTCTCCCTCACAAGGCAATGAACAAACCTCTTCCAAGAAGGGGACTCC
ACCTAAGAAGGATAAACCAACTTGTGCATATTGCAAAAAGTATGGTCATGATGAGCATCGATGCCACGCAAAGCA
AGTTGATGAGTTGACCAATCTTCTAAAGAAAAACAACATCAGCTTACCATCTACCTACATAAAGAAGGATTCATCTT
CTTCTTCTTCCTCACAGTCTAA
>gi|162316211|gb|ET185265.1
TGATAAGGGAGTATCTTTTTAATCCACCAATTTTGATGCCACCTATACAGGGTAAACCTTTGATTCTCTACATATTG
GCTATTGATACATTACTGGGGACATTTCTTGCTCAACATGATGAGAAAAAAAGCAAAGAGCTATCTATTACATCAG
TAGGACATTGGTGTCTTATGAGAAAAATTACTCCATGATCAAAAAGGCTTGCTTGGCAGTGGTCTTTGCTTCACAA
AAGTTGAGACATTACATGTTGGCATACTCTATAAAGTTGATAGCTAAGATTGATCCCTTGAAGTACCTTCTTAGCAA
AGCAACACTCATAAGATGATTGGCTAAGTGGGTAATGATTCTTATAGAGTTCAATATTGAGTATGTGGAATGAAA
GGCTATAAAAGGGTAGGCCATTGTAGATCAGCTTGCTAGTGCTCCACTTACTAATGATCAACCATTGCACATTGAG
TTTCTAGATGAGTCTAA
>gi|162316207|gb|ET185261.1
TGTTGTACTAATTTACTGGATAATAAGAAAAGATTGGATGACTATGTTGAGTGGATGTAGCCCATCTTGGGTGAAC
GATGTTAAATCTCTATTATTTTTGTGTTGTGTTTTTTTTCATCTTTTGTGTATTCAAATCTGCATATAATTGTTAATTTA
GATTTGCTCTAGATCTATCAAAACCCTAACAATTAGTATGAGAGCCATGTATTTTTCTAAATTGATAGGGATGCTTA
GGTTTGAGTGGGAGCAATGGAAGAAACCAAATTCAAGGTCAAAAATTTCAATCGCCAAAATTATCAATTATGGAA
AATGCAGATGGAGGATTACTTGTACCAAAAGGATCTATGGAAGCAATTGGAAGGAAAGTCAAAGAAACTAACCAT
GATGTCAGATGAAA
>gi|162316195|gb|ET185249.1
TAGGGCAGCAGAAATGCAGCACCTCAGCAGCACCCAGCAGCACTTTTGCAGCACTTTTATGCCTTATCAGCAGCAG
ACAGCAGCACTTTTGCAGCACTTTGAATTCACCTACTATTCTCTGAACACGGCTAATCTCTCCCTCCTGAACACCCCG
GGTCCTACTATTTATTTGCCTGCACGGGGCCGGGAGGAGCCTTCGCATCCCTTCCCGTCCTATAGTGACCGCATCTC
GATTCTATACGTAACATGCCAACCCCCTACCCCTATACGGCTATTTTGAAGGGGCAGGTAAGGTAAGGTAAGGTAA
GGTAGGCAAGATGAGGGATTACCTTAGAATAGTAGGGGCCATAGAATAGTAGTAGGGGCCCACCCATGTTACGT
ATAGAATCTGCCCCCAATAATAGA
