# Supplementary material for: Characterization of the genome of bald cypress
Source: BMC Genomics. 2011 Nov 11;12:553. doi: 10.1186/1471-2164-12-553 (PMC3228858; doi:10.1186/1471-2164-12-553)
Supplement: Additional file 2 — Primers and overgos used in macroarray analysis. [file 1471-2164-12-553-S2.PDF]

Primers and overgos used in macroarray analysis

Table A1 – Primers used in generation of gene-specific PCR amplicons used as probes in macroarray analysis

Gene	Gene abbreviation	GenBank Accession	Primer sequence, forward	Primer sequence, reverse	Tm (°C)	Product size (bp)
Putative ammonium transporter	*AMT*	AB211839	ATTAGCTTTGCCCCGTCTCT	TGCATGCCATCTCCATAAAA	52	500
Aquaporin	*AQU*	AB211841	AGTTCAGGAATGGGGACACA	ATGACGACAGGTGCTGACTG	56	499
Calmodulin	*Cal*	AB211840	AGGGAATTGAAGCTGAAGCA	GCCAAAAGGCTACAACCAAA	54	495
Pollen allergen	*Cryj2*	AB211842	GCATTCTCGTCATGCTGCTA	CTTGCTGGATTTTGGTACGC	54	516
Class I chitinase	*Chi1*	AB096607	AAAGGCGAATGTGACGGAAAT	CTGGATGATTTGCGGTGTCT	55	1172
Ferredoxin*	*Ferr*	AB096608	TCTGCGGCTGTAGTTCCAGT	ATAGGCGACGCAGGTCAAAA	57	345
Glutamyl-tRNA reductase*	*HemA*	AB161815	CTTCGGCAGCATCTCTTCAT	AACCTTAGCCCTGATTTTCT	51	1047
Lycopene beta cyclase*	*Lcyb*	AB096608	ATGGAGTTTGGGTCGATGAG	ATTGGCAGAAAAAGGCATTG	55	506
Phosphoribosylanthranilate transferase*	*Pat*	AB161910	ATGTGTTGTCTGGCTTGGTA	GGTGAACAAGAAAGGGAAAT	50	758

*Primers were not designed by us from GenBank sequence but rather were obtained from Kado et al. (2006)

Table A2 – Overgos designed from bald cypress genes used as probes in macroarray analysis

Gene	Gene abbreviation	GenBank Accession	Overgo sequence, forward	Overgo sequence, reverse
Lycopene beta cyclase	*Lcyb*	AB096608	AATAGGAGGAACTGCAGGAATG	GTTGATGGGTGCACCATTCCTG
Class I chitinase	*Chi1*	AB096607	GCAGGGTCCTTGCGCTTCTGGA	CCATAATATTGCTTTCCAGAAG
Ferredoxin	*Ferr*	AB096608	TTGCGGGGAATGTAGAGATGGA	AGAAGCTCTGATCCTCCATCTC
Glutamyl-tRNA reductase	*HemA*	AB161815	GGCAGTTTGATGCATGGAGAGA	CCGTCTCCAAAGAATCTCTCCA
Phosphoribosylanthranilate transferase	*Pat*	AB161910	TACCCTTATGGGAGGAGTGACA	CTGAGTCCAAGAACTGTCACTC

Table A3 – Overgo designed from Cot-filtered bald cypress sequence with similarity to *Ginkgo biloba copia*-like retroelement sequences

GenBank Accession	Clone address	Overgo sequence-forward	Overgo sequence-reverse
ET185333	1E16	GACTAAGGAAATCTCTCTATGG	GGGCCTGCTTAAGGCCATAGAG
