# Supplementary material for: Characterization of the genome of bald cypress
Source: BMC Genomics. 2011 Nov 11;12:553. doi: 10.1186/1471-2164-12-553 (PMC3228858; doi:10.1186/1471-2164-12-553)
Supplement: Additional file 3 — Isolation of nuclear DNA from plants. This is the standard Mississippi Genome Exploration Laboratory (MGEL) nuclear DNA isolation protocol used to obtain the milligram quantities of plant nuclear DNA required for Cot analysis and Cot filtration. [file 1471-2164-12-553-S3.PDF]

MISSISSIPPI GENOME EXPLORATION LABORATORY
Director: Daniel G. Peterson
www.mgel.msstate.edu

ISOLATION OF NUCLEAR DNA FROM PLANTS

Based on Peterson et al. (1997), Plant Mol. Biol. Reptr. **15**: 148-153.

This is an extremely robust protocol that has been used to isolate highly-pure milligram quantities of nuclear DNA from a wide variety of plants including pine, tomato, juniper, cypress, sorghum, maize, soybean, cycad, fern, prickly pear cactus, cabbage, and cotton. It is specially designed for plants in which polyphenols are a problem, although it has provided good results for every plant species on which it has been tested. If properly conducted, this protocol provides nuclear DNA that (a) has little or no visible coloration, (b) possesses a spectrophotometric $(A_{260}-A_{320})/(A_{280}-A_{320})$ value > 1.7, (c) has a mean fragment length between 50 and 100 kb, and (d) is readily digestible with *Hind*III or other common restriction endonucleases. Of note, the protocol can be used to isolate DNA from adult plant leaves as well as younger tissues (including seedlings). The protocol works best if the tissue is fresh. It generally does not work well with frozen tissue or lyophilized tissue (these processes commonly rupture organelles including nuclei). If one does not have access to fresh tissue, leaves/seedlings can be sent from a remote location *via* overnight mail. For best results, the tissue should be wrapped in damp paper towels, sealed in plastic bags, and shipped on ice.

The protocol has been successfully adapted for use in BAC library construction (see http://www.mgel.msstate.edu/newbac.htm for specifics).

EXPERIMENTAL PROCEDURES

I. *MATERIALS*

(1) *Diethyl ether* (4°C)

(2) *MEB (MPD-based Extraction Buffer)* (MADE DAY OF EXPERIMENT): This medium is specifically designed to prevent polyphenolic compounds (common in plants) from interacting with DNA. When oxidized, polyphenols covalently bind to DNA making it useless for most research applications. Additionally, MEB inhibits DNase activity. The contents of the medium are as follows: 1.0 M 2-methyl-2,4-pentanediol (Aldrich, cat. no. 11,210-0), 10 mM PIPES-KOH, 10 mM $MgCl_2{\bullet}6H_20$, 2% polyvinylpyrrolidone (PVP) (Sigma, cat. no. PVP-10), 10 mM sodium metabisulfite, 5 mM 2-mercaptoethanol, 0.5% sodium diethyldithiocarbamate (Sigma, cat. no. D-3506; store at -20°C with dessicant), 200 mM L-lysine, and 6 mM EGTA, pH 6.0. Prepare MEB on the day of the extraction, and chill the buffer to 4°C before starting the isolation. To make 3000 ml of MEB (enough for a typical experiment), place the following into a 4000 ml plastic beaker:

- 354.54 g of 2-methyl-2,4-pentanediol (stabilizes organelles)
- 9.07 g of PIPES-KOH (common component of plant growth media)
- 6.10 g of $MgCl_2{\bullet}6H_20$ (necessary for stabilization of nuclei)
- 5.70 g of sodium metabisulfite (antioxidant, prevents oxidation of polyphenols)
- 15 g of sodium diethydithiocarbamate (antioxidant, prevents oxidation of polyphenols)
- 2000 ml of distilled water
- 1.2 ml of 2-mercaptoethanol (antioxidant, prevents oxidation of polyphenols)
- 87.7 g L-lysine (DNase inhibitor)
- 6.8 g EGTA (DNase inhibitor)

Mix using a stir bar. Add 60 g of PVP (Sigma, cat. no. PVP-10) a little at a time to prevent formation of PVP clumps (PVP adsorbs polyphenols thus preventing them from interacting with DNA). Add highly concentrated HCl to the medium until the pH is between 3 and 4 and is not drifting upwards. Add NaOH pellets and/or aqueous NaOH (e.g., 1N NaOH) until the pH is 6.0 (low pH

inhibits the enzyme polyphenol oxidase). Add crushed ice until the total volume of the mixture is 3000 ml. Place the mixture in a refrigerator (cold temperatures inhibit most enzymes including polyphenol oxidase and DNases). Cover the beaker with plastic wrap. If possible, use a stir plate and stir bar to mix the solution as it sits in the refrigerator (this helps make the solution "uniformly" cool).

(3) *MPDB (2-methyl-2,4-pentanediol buffer)* (MADE DAY OF EXPERIMENT): 0.5 M 2-methyl-2,4-pentanediol (Aldrich, cat. no. 11,210-0), 10 mM PIPES-KOH, 10 mM $MgCl_2 \cdot 6H_20$, 0.5% Triton X-100, 10 mM sodium metabisulfite, 5 mM 2-mercaptoethanol, 200 mM L-lysine, and 6 mM EGTA, pH 7.0. To make 50 ml of MPD buffer (enough for a typical experiment), place the following into a 100 ml beaker:

- 2.95 g of 2-methyl-2,4-pentanediol (stabilizes organelles)
- 0.15 g of PIPES-KOH (common component of plant growth media)
- 0.10 g of $MgCl_2 \cdot 6H_20$ (prevents nuclei from lysing prematurely)
- 0.10 g of sodium metabisulfite (prevents oxidation of polyphenols)
- 0.25 ml of Triton X-100 (detergent that lyses chloroplasts and mitochondria)
- 19.5 µl of 2-mercaptoethanol (antioxidant)
- 1.5 g of L-lysine (inhibitor of DNases)
- 0.11 g of EGTA (inhibitor of DNases)

Bring the total volume to 50 ml with distilled/deionized water. Add NaOH until the final pH is 7.0. Place the solution in a refrigerator.

(4) *Cheesecloth*

(5) *Miracloth*

(6) *100X TE (Tris-EDTA) stock buffer*: 1.0 M Tris base (also known as THAM), 0.1 M EDTA. To make 2 L, place 242.28 g of Tris-base and 58.44 g of EDTA (or 74.44 g of EDTA disodium salt: dihydrate) in a 4 L beaker. Add distilled water to 1.5 L. Adjust pH with concentrated HCl to yield a solution that is pH 7.0. Add distilled water to a final volume of 2 L. Place the 100X TE in glass bottles.

(7) *1X TE (non-sterile)*: Dilute the 100X TE stock solution 1:100 with distilled water.

(8) *3 M sodium acetate* (aqueous, pH 5.2) - This solution can be stored in 50 ml polypropylene tubes at -20°C.

(9) *70% v/v and 100% v/v ethanol* (-20°C)

(10) *Proteinase K* (molecular biology grade)

(11) *RNaseA stock solution*: Mix RNaseA with 1X TE to yield a 20,000 µg/ml RNaseA solution. Boil the solution in a closed container for 10 min to destroy any contaminating DNases. Mix with an equal volume of glycerol to produce a 10,000 µg/ml stock solution. Store in 1 ml aliquots at -20°C. Vortex before using.

(12) *RNase T1 stock solution*: Mix RNaseT1 with 1X TE to yield a 20,000 unit/ml RNaseT1 solution. Boil the solution in a closed container for 10 min to destroy any contaminating DNases. Mix with an equal volume of glycerol to produce a 10,000 unit/ml stock solution. Store in 1 ml aliquots at -20°C. Vortex before using.

(13) *Methylene blue* (1.0% w/v - aqueous)

(14) *20% v/v SDS (sodium dodecyl sulfate)*

(15) *5 M sodium perchlorate* (aqueous)*:* Store at -20ºC.

(16) *Kitchen blender*

(17) *Light microscope*: The microscope should be capable of bright-field and/or phase-contrast illumination and have a total magnification power of at least 200x.

(18) *Dialysis tubing* (12,000-14,000 MWCO): For example, Spectrum Spectra/Por 2 Tubing, cat. no. 132678. This item is available from both VWR and Fisher Scientific.

(19) *Dialysis tube closures*: For example, Spectrum Spectra/Por Weighted and Unweighted Closures, cat. no. 132751. This item is available from both VWR and Fisher Scientific.

(20) *Buffer-saturated phenol (pH 7.9)*

(21) *Chloroform*

(22) *Isoamyl alcohol*

II. *METHODS*

DAY 1 - *Preparation*

(1A) Place 4000 ml of 1X TE in each of three polypropylene (or glass) beakers. The 1X TE is prepared from the 100X TE stock solution (see MATERIALS above). To produce 4000 ml of 1X TE from the 100X TE stock solution, simply place 40 ml of stock solution in each beaker and add distilled water until the final volume in each is 4000 ml. Place these beakers of 1X TE in a refrigerator.
(1B) Place 2 L of ether in a refrigerator or freezer if it is not already stored in the cold.
(1C) Make sure 20% SDS, 3 M sodium acetate, 5 M sodium perchlorate, and 1.0% methylene blue solutions are prepared and ready for use. Also be sure to make up RNase A and RNase T1 stock solutions if they have not already been prepared (see instructions in MATERIALS above).
(1D) DO NOT MAKE UP THE MEB OR MPDB UNTIL THE DAY OF THE EXPERIMENT (these solutions lose their potency if stored for more than a few hours at 4°C). In our experience, storing MEB and MPDB in a freezer also results in a loss of effectiveness.

DAY 2 - *Homogenization, filtration, extraction, and dialysis*

(2A) Prepare MEB and MPDB as described in the MATERIALS section above. Ideally, the medium is prepared before any tissue is harvested (*i.e.*, the fresher the tissue, the better the results).
(2B) Collect tissue.

(i) For mature plants, young leaves or needles are gently picked from the plant of interest. After removing a leaf/needle, it is immediately submerged in ice cold 1X TE (use one of the beakers of 1X TE prepared the day before). Approximately 200-1000 g of leaves/needles are collected for one isolation.

(ii) For DNA isolation from seedlings, plant seeds at high density in soil in greenhouse trays, cover seeds with 0.5 cm of soil, water, and place trays in the greenhouse. At the time of harvest, cut the tops from the plants and submerge the tops in ice cold 1X TE (use one of the beakers prepared the day before). The optimum time of harvest depends on the plant. For monocots, allow seedlings to grow to a height of approximately 20-30 cm before harvesting. For dicots, wait until true leaves appear and flourish before harvesting as cotyledons are senescent tissue and do not yield good quality DNA. Approximately 500-100 g of seedlings are collected for one isolation.

(2C) Remove leaves/needles/seedlings from the 1X TE slurry and place them in 1000-3000 ml of ice-cold diethyl ether (*i.e.*, enough to submerge all tissue) for three minutes (PERFORM THIS STEP IN A FUME HOOD!). Ether removes waxes and makes cells more friable. Remove leaves/needles/seedlings from the ether and then wash them two times (2 min each wash) in 4000 ml 4°C 1X TE (use remaining beakers prepared on the day before). Place the leaves/seedlings in 3000 ml of ice cold MEB. Homogenize leaves/needles/seedlings in the MEB using a kitchen blender (highest speed attainable for 30 seconds). Squeeze the homogenate through six layers of cheesecloth. Then filter the resulting filtrate through 32 layers of cheesecloth. If possible, let this second filtration occur by gravity only (*i.e.*, no squeezing).
(2D) Add Triton X-100 to the filtrate to a final concentration of 0.5% v/v. Fill four 500 ml centrifuge bottles with the mixture and spin at 800 x *g* for 20 min at 4°C. Place the remaining mixture in the refrigerator. Decant supernatants, place the remaining homogenate in the bottles, and centrifuge as described above (this reduces the number of bottles one has to wash at the end of the experiment). At 0.5% v/v, the Triton X-100 preferentially lyses chloroplasts and mitochondria while leaving nuclei more or less intact.
(2E) Resuspend each pellet by vortexing in 0.5 ml of MPDB, and pool suspensions. In most species pellets will go into suspension fairly easily. However, in some (like tomato),

extensive vortexing is necessary; likewise, the pellet may need to be agitated with a spatula to get it unstuck from the bottle wall. In some species, there may be some clumps of green material (presumably chlorophyll) that will remain in clumps despite vigorous mixing. These clumps can be filtered out of the solution by passing it through a couple of layers of Miracloth.
(2F) Mix a small drop of the suspension with an equal volume of 1.0% methylene blue on a microscope slide. Add a coverglass and examine the slide by phase-contrast and/or bright-field microscopy. If all has gone well, the mixture should contain numerous dark-blue stained nuclei, no intact cells, and no mitochondria or chloroplasts. Starch grains (visible by phase-contrast microscopy) should stain little, if at all, with methylene blue (see **FIGURE 1**).
(2G) Add 10 ml of MPDB, and transfer the nuclear suspension to a 15 ml polypropylene centrifuge tube. Take a tube of 5 M sodium perchlorate out of the freezer so that it can thaw and warm to room temperature.
(2H) Centrifuge at 300 x *g* for 10 min, and then turn the centrifuge up to a speed equivalent to 650 x g.
Allow the tube to spin for an additional ten minutes. Discard the supernatant.
(2I) The resulting pellet may or may not contain two or more distinct layers. If there is a single layer, proceed to step 2L. If not, perform steps 2J and 2K.
(2J) If more than one layer is present, add two ml of MPDB and gently vortex the tube until the upper-most layer is resuspended. Pour this suspension into its own 15 ml centrifuge tube leaving the rest of the pellet in the first tube.
(2K) Repeat step 10 until the original pellet layers are roughly segregated into different tubes. Prepare a slide from the contents of each tube (see step 2F). Based on the microscopy results, pick the tube(s)/layer(s) that contains the most nuclei and the least debris. Discard the other tubes.
(2L) Add 10 ml of MPDB to the nuclei and mix by gentle inversion. Centrifuge the suspension at 650 x *g* for 10 minutes, and carefully decant the supernatant. Highly pure nuclei do not form a very hard pellet and may slide out of the tube if the supernatant is discarded too forcefully.
(2M) Gently tap the tube so that the nuclei become resuspended in the residual MPDB left in the tube. If necessary, add a little more MPDB to get the nuclei in suspension. HOWEVER, YOU WILL WANT TO KEEP THE TOTAL VOLUME AS SMALL AS POSSIBLE! In our experience, keeping the volume to a minimum increases yield and reduces DNA fragmentation.
(2N) Add 20% aqueous SDS (w/v) to a final concentration of 2% (w/v). Mix contents of the tube by gentle inversion to lyse the nuclei.
(2O) Heat the tube containing the nuclear lysate in a water bath at 60°C for 10 min, cool to room temperature, and add 5 M sodium perchlorate (20°C) to a final concentration of 1 M. Sodium perchlorate breaks up many protein/DNA interactions.
(2P) Spin the lysate in a swinging bucket rotor at 400 x *g* for 20 min to pellet starch grains. Transfer the supernatant to a sterile polypropylene tube using a 1000 µl plastic pipet tip from which the bottom third has been cut off. Perform all subsequent transfers of DNA-containing solutions using such modified pipet tips to minimize shearing of the DNA.
(2Q) Perform a DNA extraction by mixing the nucleic acid solution with an equal volume of phenol/chloroform/isoamyl alcohol (25:24:1). To minimize shearing of DNA, use a test tube rocker (18 cycles per min for 30 min) rather than a shaker to mix the organic and aqueous phases. Centrifuge the mixture at 3000 x *g* in a swinging bucket rotor for 10 min. Transfer the upper aqueous phase into a new sterile polypropylene tube.
(2R) Perform a second extraction as described above.
(2S) Dialyze the aqueous phase (containing nucleic acids) into TE (pH 7.0) at 4°C (see the MGEL protocol "Dialysis of Nucleic Acid Solutions" for details).

DAY 3 – *RNase & proteinase digestions, extractions, and precipitation*

(3A) Place the dialyzed sample into a 15 ml polypropylene tube or a microfuge tube (depending upon sample volume).
(3B) Vortex the RNase T1 and RNase A stock solutions. Add RNase T1 and RNase A stock to the sample so that their final concentrations in the solution are 50 units/ml and 50 µg/ml, respectively. Mix the sample by inverting the tube 10-20 times. Incubate at 37°C for 60 min.
(3C) Make the solution 150 µg/ml Proteinase K, and incubate at 37°C for 60 min.
(3D) Perform two additional phenol/chloroform/isoamyl alcohol extractions followed by two extractions using only chloroform. Transfer the aqueous phase to a microcentrifuge tube(s), and add one-tenth volume of 3M sodium acetate (pH 5.2). Mix by inverting the tube several times.
(3E) Add two volumes of ethanol and mix thoroughly. The DNA will precipitate. If desired the DNA can be stored in this solution at -20°C indefinitely.
(3F) Spin the resulting mixture in a centrifuge at 14,000 rpm for 30 min at 4°C.
(3G) Gently decant the supernatant. Add 1 ml of 70% ethanol and centrifuge at 14,000 rpm for an additional 15 min. Decant the supernatant and allow the pellet to air dry for 1 hr. Alternatively, pour off the supernatant, place the tube in a vacuum centrifuge, and spin for 5-10 min until there is no trace of liquid remaining in the tube.
(3H) Redissolve the DNA in a buffer of choice.

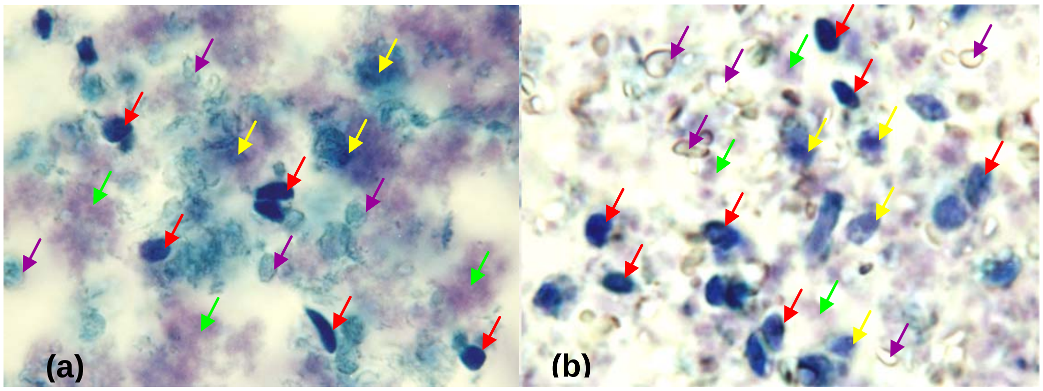

FIGURE 1 - Nuclei prepared using from **(a)** *Sorghum bicolor* (grain sorghum) and **(b)** *Solanum lycopersicum* (tomato). The preparations have been stained with methylene blue and photographed using bright-field microscopy. The nuclei stain dark blue and are variable in shape (though most are roughly spherical). Within a picture, much of the observed variation in nucleus size and shape is attributable to the fact that not all of the nuclei lie in the same focal plane. Nuclei within the focal plane (*e.g.*, red arrows) exhibit greater definition than nuclei above or below the focal plane (*e.g.*, yellow arrows). Starch grains (*e.g.*, purple arrows) often pellet with nuclei. However, unlike nuclei, starch grains stain only lightly (if at all) with methylene blue, and tend to have a refractile appearance (especially when viewed by phase-contrast microscopy). Much of the starch can be eliminated from nuclear preps by performing successive low-speed centrifugations as described in the *METHODS*. In most species we examined, some of the debris that pellets with nuclei stains lightly with methylene blue (light purple regions, *e.g.*, green arrows). Magnification = roughly 1000x.
