# Supplementary material for: Characterization of the genome of bald cypress
Source: BMC Genomics. 2011 Nov 11;12:553. doi: 10.1186/1471-2164-12-553 (PMC3228858; doi:10.1186/1471-2164-12-553)
Supplement: Additional file 4 — Shearing DNA into 450 bp fragments using the Misonix Sonicator 3000. This is a detailed MGEL protocol describing the method used to shear Taxodium distichum nuclear DNA for use in Cot analysis and Cot filtration. [file 1471-2164-12-553-S4.PDF]

MISSISSIPPI GENOME EXPLORATION LABORATORY
Director: Daniel G. Peterson
www.mgel.msstate.edu

# SHEARING DNA INTO 450 BP FRAGMENTS USING THE MISONIX SONICATOR 3000

Traditionally, Cot analysis has been performed using DNA fragments of roughly 450 bp (Britten et al. 1974; *Methods in Enzymol*. **29**: 363-405). In addition, the 454/Roche Applied Systems Genome Sequencer 20 (GS20) works most efficiently when loaded with DNA fragments between 400-500 bp (J. Carlson, personal comm.). Consequently, MGEL has a need to reproducibly generate DNA fragments with a mean length of 450 bp for Cot analysis and 454-based sequencing. Towards this end, we currently use a Misonix Sonicator 3000 (www.misonix.com). This instrument is easy to use and yields consistent results (when properly maintained) with little DNA loss. While DNA also can be sheared into pieces of 450 bp or less with a high speed blender (*e.g.*, a Virtis homogenizer) or a nebulizer, in our experience these instruments result in an unacceptable amount of DNA loss (usually > 50%). The GeneMachine's HydroShear can shear DNA with almost no loss, but we have found it incapable of shearing DNA into fragments with mean lengths < 1.0 kb (even when using special small-aperture shearing assemblies).

The following protocol describes how we currently generate 450 bp DNA fragments using the Sonicator 3000. We use only the standard ½ inch tapped horn with a ½ inch diameter titanium tip. We have not tried to use microtips. Our attempts at using a large cup horn have been unsuccessful (albeit limited). If the DNA is "pre-sheared" to 1500 bp using a HydroShear, the range in fragment size after sonication is narrower than if the DNA is not presheared.

## EXPERIMENTAL PROCEDURES

### I. *MATERIALS*

(1) *Unsheared DNA or DNA that has been presheared to ca. 1500 bp using a GeneMachines HydroShear*
(2) *50 ml glass beaker*
(3) *Misonix Sonicator 3000:* Comes with generator, converter, standard ½ inch tapped horn, titanium horn tip (½ in. diameter), temperature probe, converter cable, wrench set, and power cord. Occasionally the titanium horn tip may need to be replaced (Misonix cat. no. 406). Likewise, if the horn is dropped or injured or significantly damaged, a replacement can be obtained from Misonix (cat. no. 200).
(4) *70% v/v ethanol*
(5) *1X TE (sterile)*: 10 mM Tris-HCl (pH 8.0), 1 mM EDTA
(6) *Plastic dish approximately 3.0 cm in height.* We use the lid from a 20-200 µl pipet tip box for this purpose.
(7) *Large support (ring) stand, one large right-angle clamp, one small right-angle clamp, one large three-pronged clamp, and one small three-pronged clamp* (see **FIGURE 1** below).
(8) *Millipore Centriplus YM-30 Centrifugal Filter Unit* (cat. no. 4422)
(9) *100 bp DNA ladder* (New England BioLabs, cat. no. N3231L)

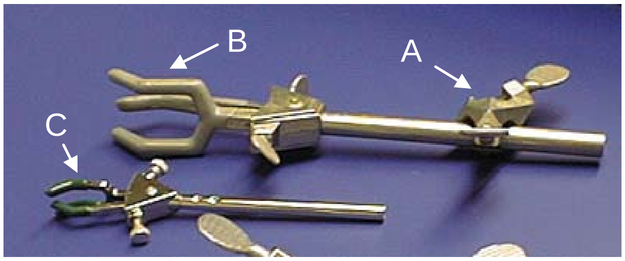


**FIGURE 1**. Some clamps used with the Sonicator 3000. (A) Large right-angle clamp. (B) Large three-pronged clamp. (C) Small three-pronged clamp. A small right-angle clamp is not shown.

## II. *METHODS*

(1) READ THE SONICATOR 3000 INSTRUCTION BOOKLET BEFORE DOING ANYTHING ELSE!

(2) Make sure that the tapped horn is tightly fastened to the converter and that the titanium tip is tightly fastened to the end of the horn (see **FIGURE 2A** for diagram of instrument). Tightening should be performed with the special wrenches provided with the instrument according to the instrument's instruction booklet. Check the titanium tip for cracks or other signs of damage, and replace as needed (see manufacturer's instructions).

(3) Set up the Misonix Sonicator 3000 as shown in **FIGURE 2A**. Use a large three-pronged clamp connected to a support stand *via* a right-angle clamp to hold the sonicator in place. Use a small three-pronged clamp to hold a 50 ml beaker securely within a plastic box resting on the base of the support stand. Wash the narrow part of the horn and the tip with deionized water, rinse with 70% v/v ethanol, and dry with a Kimwipe.

(4) Place the DNA solution in the clamp-secured 50 ml glass beaker. Add 1X TE until the final volume is 10 ml.

(5) When the titanium tip is tightly screwed into the end of the tapped horn, the visible portion of the tip extends roughly 3.5 mm past the end of the tapped horn (**FIGURE 2B**). Carefully lower the converter/horn by releasing the tension on the large right-angle clamp. SUPPORT THE CONVERTER/HORN SO THAT IT DOES NOT FALL! Lower the converter/horn so that the uppermost visible portion of the titanium tip is 5 mm below the surface of the solution (**FIGURES 2B** and **2C**). Secure the converter/horn in place by tightening the large right-angle clamp. Be aware that tightening the clamp may change the position of the tip in the liquid, so some adjustment may be required to get the tip to the recommended depth. The long axis of the horn should be perpendicular to the surface of the DNA solution and the horn should be centered in the middle of the beaker. NO PART OF THE HORN OR THE TIP SHOULD TOUCH THE BEAKER! IF THE TIP COMES INTO CONTACT WITH THE BEAKER DURING OPERATION, THE TIP AND BEAKER WILL BE DAMAGED!

(6) Once the converter/horn as at the correct height for shearing, use a black marker to draw a line on the support stand immediately below the position of the large right-angle clamp. This line can be used to quickly set the height of the converter/horn during future runs if the converter/horn is not removed from or repositioned in its three-prong clamp.

(7) Turn on the Sonicator 3000 (the on-switch is located on the back of the generator). After its initial welcome message, the screen on the generator will read:

**Are you using a MicroTip (Y/N)?**

Press the "Clear/No" button.

(8) Program the Sonicator 3000 according to the instructions that come with the unit. Once a program for shearing DNA to 450 bp is established, that program can be saved and accessed later. We have found that a program with the following values reproducibly gives us fragments with a mean length of 450 bp (or thereabouts).

<u>PROGRAM</u>

Total Process Time – 1-3 min (see **FIGURE 3**)
Pulse-ON Time – 10 sec
Pulse-OFF Time – 1 min
Initial Output Level – 2.0
Temperature Monitoring? – Yes
Temperature Control? – Yes

Max Temperature for Shutdown – 25°C
Controlling an External Device? – No

**NOTE**: WHILE THESE PARAMETERS CURRENTLY GIVE US GOOD RESULTS USING OUR SONICATOR 3000, IT IS LIKELY THAT THE PARAMETERS MAY NEED TO BE RE-OPTIMIZED FROM TIME TO TIME. LIKEWISE, IF A PART OF THE SONICATOR HAS BEEN REPLACED OR THE INSTRUMENT HAS NOT RECENTLY BEEN USED FOR SHEARING DNA TO 450 BP, PERFORM A TEST RUN USING LAMBDA OR *E. COLI* DNA TO MAKE SURE THAT THE PARAMETERS ABOVE YIELD FRAGMENTS OF AN APPROPRIATE LENGTH!

(9) Place ice in the plastic box so that the beaker is cooled during sonication (**FIGURE 2D**). Insert the temperature probe into the DNA solution. MAKE SURE THAT THE TEMPERATURE PROBE DOES NOT TOUCH THE HORN OR ITS TITANIUM TIP!
(10) Start the shearing program. If the tip is properly submerged and the tip and horn properly tightened, a faint high-pitched whirr will be heard and the liquid will move little. If the tip is not submerged, loud, high piercing sounds will eminate from the instrument and/or the solution may foam. If the piercing noise is heard or significant foaming occurs, stop the instrument and readjust the height of the converter/horn. If this does not remedy the problem, the titanium horn tip may be damaged.
(11) At the end of the program, remove the converter/horn from the beaker by loosening the large right-angle clamp. SUPPORT THE CONVERTER/HORN WHEN RELEASING THE TENSION ON THE LARGE RIGHT-ANGLE CLAMP. IF THE CONVERTER/HORN SLIDES RAPIDLY DOWN THE SUPPORT POLE AND HITS THE BOTTOM OF THE BEAKER BOTH THE TIP AND THE BEAKER MAY BE BROKEN! Slide the clamp (with attached converter/horn) up the pole and firmly tighten the clamp to secure the converter/horn.
(12) Remove the beaker containing the sheared DNA from the apparatus. Rinse the horn and tip with 70% ethanol. Submerge the tip in a beaker of 70% ethanol and run the instrument on low power (Output Level of 2 or less) for 30 sec. Dry the horn and tip with a Kimwipe. Throw out the ice and clean the area around the Sonicator 3000. Do not remove the sonicator from its three-pronged clamp unless necessary. If the clamp is not removed, the converter/horn can be quickly positioned at the appropriate height for another sonication run (using the conditions and items described above) by moving the large right-angle clamp so that it is positioned immediately above the black marker line placed on the pole of the support stand.
(13) Run the DNA solution through a Chelex column as described in the MGEL protocol "Removing Metal Ions from DNA Solutions Using Chelex."
(14) Concentrate the DNA sample and/or perform a buffer exchange using a Centriplus YM-30 column (see manufacturer's instructions).
(15) Determine the concentration of the concentrated DNA sample by spectrophotometry. Run 0.5 µg of the sheared DNA on a 1.0% agarose gel against the 100 bp DNA ladder. The DNA should have a mean fragment size of 450 bp (or thereabouts) (**FIGURE 3**).

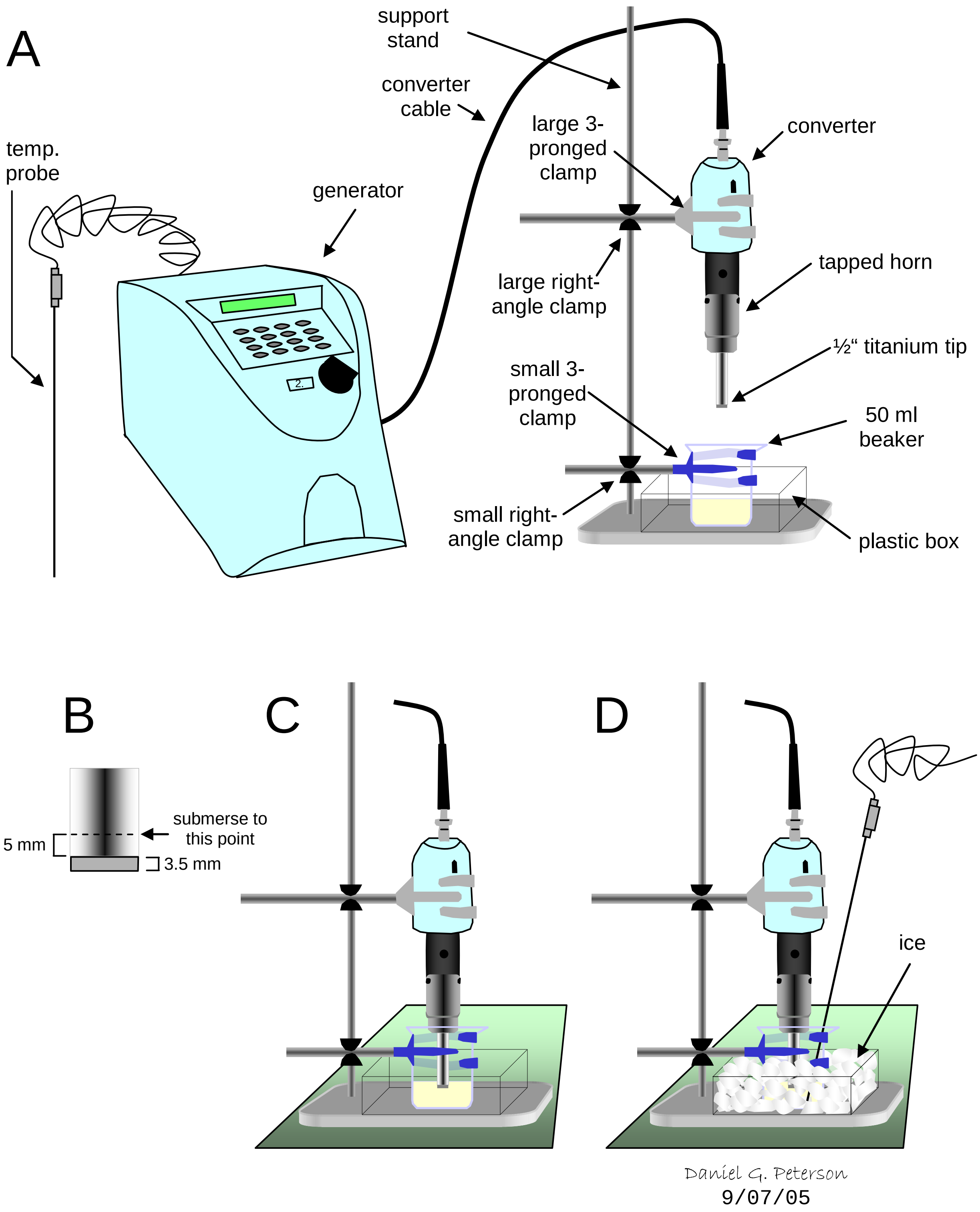


**FIGURE 2**. Using the Sonicator 3000 to produce 450 bp DNA fragments. See the text for explanation.

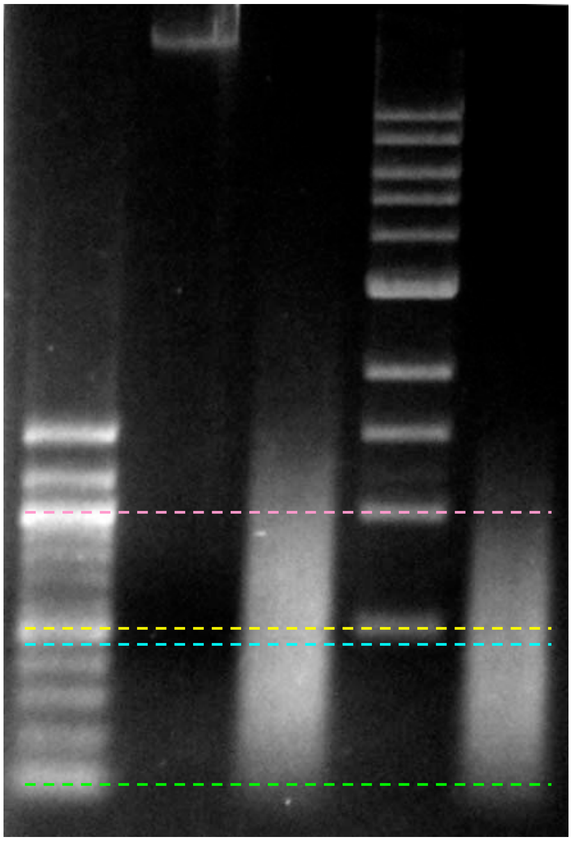

**FIGURE 3**. DNA sheared using the Misonix Sonicator 3000 with the parameters described in the text. Lane 1 (far left) contains the New England BioLabs 100 bp ladder, lane 2 contains unsheared *E. coli* DNA, lane 3 contains *E. coli* DNA sheared using a total process time of 1 min, lane 4 contains the New England BioLabs 1 kb ladder, and lane 5 contains *E. coli* DNA sheared using a total process time of 2 min. Dotted lines mark locations of 1000 bp (pink), 500 bp (yellow), 100 bp (green), and 450 bp (turquoise) fragments. Both the 1 and 2 min total processing times yield DNA fragments with mean lengths near 450 bp. The sample with the 2 min process time shows less variation in fragment size, although the mean fragment size is slightly < 450 bp.
