# Supplementary material for: Characterization of the genome of bald cypress
Source: BMC Genomics. 2011 Nov 11;12:553. doi: 10.1186/1471-2164-12-553 (PMC3228858; doi:10.1186/1471-2164-12-553)
Supplement: Additional file 5 — Removing metal ions from DNA solutions using Chelex. This is a detailed MGEL protocol describing removal of metal ions from DNA solutions using Chelex. [file 1471-2164-12-553-S5.PDF]

MISSISSIPPI GENOME EXPLORATION LABORATORY
Director: Daniel G. Peterson
www.mgel.msstate.edu

REMOVING METAL IONS FROM DNA SOLUTIONS USING CHELEX

It is imperative in Cot analysis that metal ions are removed from DNA solutions prior to denaturation and renaturation. If metal ions are left in a DNA solution they can cause a number of problems. For instance, some metal ions can activate DNases which may be present in trace amounts even after multiple phenol-chloroform extractions. Additionally, superoxide radicals and hydrogen peroxide can be produced in any solution in which O_2 is dissolved as long as there is some type of metal ion around. The production of reactive oxygen species is exacerbated if a solution containing metal ions is heated above room temperature (Bruskov et al. 2002; *Nucl. Acids Res.* **30**: 1354-1360). Superoxide radicals and hydrogen peroxide can depurinate DNA and thus cause mutations that will confuse the results of Cot-Based Cloning and Sequencing.

Chelex resin is one of the most effective means by which metal ions can be removed from DNA solutions. It is also the means by which the original practitioners of Cot analysis eliminated metal ions from their DNA solutions (Britten et al. 1974; *Meth. Enzymol.* **29**: 363-405).

EXPERIMENTAL PROCEDURES

I. *MATERIALS*

(1) *Chelex 100* (50-100 mesh, sodium form, Bio-Rad Cat. No. 142-2822): Chelex resins contain paired iminodiacetate ions coupled to a styrene divinylbenzene support. They are unique chelating resins that bind polyvalent cations with high selectivity and are used to remove metal ions from samples and buffers.
(2) *3 M sodium acetate* (pH 7.0) – Store in 50 ml aliquots at -20°C.
(3) *10-50 ml syringe* (sterile)
(4) *Glass wool* (autoclaved)
(5) 0.06 M sodium acetate (pH 7.0): Make 200 ml by mixing 4 ml of 3 M sodium acetate (pH 7.0) with 196 ml of deionized water.
(6) *Millipore Centriplus YM-30 Centrifugal Filter Units* (Cat. No. 4422). See http://www.millipore.com/catalogue.nsf/docs/4422 for further information.

II. *METHODS*

(1) Place 4-5 tablespoons of Chelex beads in a beaker.
(2) Add 50 ml of 3 M sodium acetate (pH 7.0) to the Chelex in the beaker. Swirl gently for 30 sec, and then let the Chelex settle out of the solution (approximately 10 min). Aspirate or decant the buffer.
(3) Repeat step 2 two additional times.
(4) Add 50 ml of 0.06 M sodium acetate (pH 7.0) to the Chelex in the beaker. Swirl gently for 30 sec, and then let the Chelex settle out of the solution (approximately 10 min). Aspirate or decant the buffer.
(5) Repeat step 4 two additional times.
(6) Resuspend the Chelex in 50 ml 0.06 M sodium acetate (pH 7.0). This “equilibrated Chelex” can be stored for a several months at 4°C.
(7) Place a small plug of glass wool in the bottom of a 10-50 cc syringe (no needle). Swirl

the Chelex solution and pour some into the barrel of the syringe until a pad of Chelex of approximately 5 ml is produced. Allow the buffer to drain through the column until the buffer level is 2 mm above the top of the pad of Chelex. Stop the flow of buffer through the column (syringe) by quickly wrapping the tip of the syringe with Parafilm.

(8) Make the DNA solution 0.06 M sodium acetate by adding an appropriate amount of 3 M sodium acetate (pH 7.0). Swirl the solution.

(9) Remove the Parafilm from the tip of the syringe and place the DNA solution onto the Chelex column. Collect the eluant in a clean siliconized beaker.

(10) Once the column has stopped flowing, add an additional 5 ml of 0.06 M sodium acetate to the column to elute any remaining DNA.

(11) Place the DNA solution into a Centriplus YM-30 Centrifugal Filter Unit(s). Concentrate the DNA and exchange the buffer with an appropriate sodium phosphate buffer (*e.g.*, 0.03, 0.12, or 0.5 M SPB) using the Centriplus YM-30 instructions.
