# Supplementary material for: Characterization of the genome of bald cypress
Source: BMC Genomics. 2011 Nov 11;12:553. doi: 10.1186/1471-2164-12-553 (PMC3228858; doi:10.1186/1471-2164-12-553)
Supplement: Additional file 6 — Preparing 0.5 M sodium phosphate buffer (SPB). This is a detailed MGEL protocol describing how to prepare sodium phosphate buffer for use in Cot analysis and Cot filtration. [file 1471-2164-12-553-S6.PDF]

MISSISSIPPI GENOME EXPLORATION LABORATORY
Director: Daniel G. Peterson
www.mgel.msstate.edu

PREPARING 0.5 M SODIUM PHOSPHATE BUFFER (SPB)

Sodium phosphate buffer (SPB) has a strong buffering capacity and the effects of SPB concentration on DNA reassociation have been quantified (Britten et al. 1974; Meth. Enzymol. **29**: 363-405). SPB is the primary buffer used in Cot research, and consequently it is the buffer that we use in most of our DNA reassociation kinetics experiments.

Changes in cation concentration affect reassociation rate. Thus it is very important that SPBs of known molarity are used in Cot analysis. Sodium phosphate monobasic and dibasic powders are hygroscopic. A hygroscopic material (literally "water seeking") is one that readily absorbs water, usually from the atmosphere. If one is trying to make up a solution of a precise molarity by weighing out a particular amount of hygroscopic solute, there is a level of uncertainty as some of the weight will be contributed by atmospheric water, not the solute itself. Consequently, it is best to make up SPB using a refractometer, an instrument that allows one to determine the "exact" molarity of a solution. Because it is a hassle to make up SPB on a regular basis, we recommend making up large batches of 0.5 M SPB and storing the buffer in aliquots at -20°C. Other commonly used SPB concentrations (*e.g.*, 0.03 and 0.12 M) can be prepared by dilution of the 0.5 M SPB stock solution.

EXPERIMENTAL PROCEDURES

I. *MATERIALS*

(1) *Sodium phosphate, dibasic, anhydrous (Na_2HPO_4)* - FW = 141.96
(2) *Sodium phosphate, monobasic, monohydrate ($NaH_2PO_4 \bullet H_2O$)* - FW = 137.99
(3) *Abbe Refractometer*
(4) *Distilled deionized water (ddH_2O)*
(5) *Two clean containers capable of holding 5 L of liquid each*
(6) *One large container capable of holding 10 L of liquid*
(7) *500 ml bottles and 50 ml tubes (polypropylene) with caps*

II. *METHODS*

(1) Place 413.97 g of $NaH_2PO_4 \bullet H_2O$ in a 5 L container. Add 3 L of ddH_2O. Stir the solution until the $NaH_2PO_4 \bullet H_2O$ has gone into solution. Add ddH_2O up to 5 L and mix thoroughly. If the $NaH_2PO_4 \bullet H_2O$ has not absorbed any water from the environment, the resulting $NaH_2PO_4 \bullet H_2O$ solution should be about 0.6 M.

(2) Place 425.88 g of Na_2HPO_4 in a 5 L container. Add 3 L of ddH_2O. Stir the solution until the Na_2HPO_4 has gone into solution. Add ddH_2O up to 5 L and mix thoroughly. If the Na_2HPO_4 has not absorbed any water from the environment, the resulting Na_2HPO_4 solution should be about 0.6 M.

(3) Place a drop of pure ddH_2O on the measuring prism surface of the refractometer and measure its refractive index (n) according to the manufacturer's instructions. Pure water should have an n of 1.3330. Write down the value obtained for your deionized water sample (*e.g.*, 1.3328). Subtract the theoretical value for pure water from the measured value (*e.g.*, 1.3328 – 1.3330 = - 0.0002) to obtain a "correction value"

(CRV). Note that a CRV may be positive or negative.

(4) Clean and dry the measuring prism. Place a drop of $NaH_2PO_4 \bullet H_2O$ solution on the measuring prism and determine its *n*. Ultimately, you would like to produce a 0.5 M sodium phosphate monobasic solution. Such a solution prepared with your ddH_2O should have an *n* of 1.34045 + CRV [*e.g.*, 1.34045 + (-0.0002) = 1.34043].

  (a) If the observed *n* is higher than the desired *n*, add a small quantity of ddH_2O to the $NaH_2PO_4 \bullet H_2O$ solution, mix thoroughly, and check the *n* again. Continue this process until the *n* of the $NaH_2PO_4 \bullet H_2O$ buffer is 1.34045 + CRV.

  (b) If the observed *n* is lower than the desired *n*, add a small quantity of powdered $NaH_2PO_4 \bullet H_2O$ to the $NaH_2PO_4 \bullet H_2O$ solution, mix thoroughly (until all the powder is clearly dissolved), and check the *n* again. Continue this process until the *n* of the $NaH_2PO_4 \bullet H_2O$ buffer is 1.34045 + CRV.

(5) Clean and dry the measuring prism (with ddH_2O and a Kimwipe). Place a drop of Na_2HPO_4 buffer on the measuring prism and determine its *n*. Ultimately, you would like to produce a 0.5 M sodium phosphate dibasic solution. Such a solution prepared with your deionized water should have an *n* of 1.34580 + CRV [*e.g.*, 1.34580 + (-0.0002) = 1.34578].

  (c) If the observed *n* is higher than the desired *n*, add a small quantity of ddH_2O to the Na_2HPO_4 solution, mix thoroughly, and check the *n* again. Continue this process until the *n* of the Na_2HPO_4 solution is 1.34580 + CRV.

  (d) If the observed *n* is lower than the desired *n*, add a small quantity of powdered Na_2HPO_4 to the Na_2HPO_4 solution, mix thoroughly (until all the powder is clearly dissolved), and check the *n* again. Continue this process until the *n* of the Na_2HPO_4 solution is 1.34580 + CRV.

(6) Place exactly 5 L of the 0.5 M $NaH_2PO_4 \bullet H_2O$ solution in a > 10 L container. Add exactly 5 L of 0.5 Na_2HPO_4 solution to the 5 L of 0.5 M $NaH_2PO_4 \bullet H_2O$ solution. Mix thoroughly. The result is 0.5 M sodium phosphate buffer (0.5 M SPB).

(7) Pour the 0.5 M SPB into numerous plastic storage containers (*e.g.*, 500 ml bottles and 50 ml polypropylene tubes) so that each is no more than 75% full. Place tight sealing caps and content labels on each container. Store the containers at -20°C. In our experience, 0.5 M SPB can be stored at -20°C for at least three years (and perhaps indefinitely) without a change in efficacy.
